# Selenoprotein P-neutralizing antibodies improve insulin secretion and glucose sensitivity in type 2 diabetes mouse models

**DOI:** 10.1038/s41467-017-01863-z

**Published:** 2017-11-21

**Authors:** Yuichiro Mita, Kaho Nakayama, Shogo Inari, Yukina Nishito, Yuya Yoshioka, Naoko Sakai, Kanade Sotani, Takahiro Nagamura, Yuki Kuzuhara, Kumi Inagaki, Miki Iwasaki, Hirofumi Misu, Masaya Ikegawa, Toshinari Takamura, Noriko Noguchi, Yoshiro Saito

**Affiliations:** 1 Systems Life Sciences Laboratory, Department of Medical Life Systems, Faculty of Life and Medical Sciences,, Doshisha University, Kyoto, 610-0394 Japan; 2 Organization for Advanced Research and Education, Doshisha University, Kyoto, 610-0394 Japan; 3 Genomics, Proteomics and Biomedical Functions, Department of Life and Medical Systems, Faculty of Life and Medical Sciences,, Doshisha University,, Kyoto, 610-0394 Japan; 4 Department of Endocrinology and Metabolism, Kanazawa University Graduate School of Medical Sciences, Kanazawa, Ishikawa 920-1192 Japan

## Abstract

Selenoprotein P (SeP) functions as a selenium (Se)-supply protein. SeP is identified as a hepatokine, promoting insulin resistance in type 2 diabetes. Thus, the suppression of Se-supply activity of SeP might improve glucose metabolism. Here, we develop an anti-human SeP monoclonal antibody AE2 as with neutralizing activity against SeP. Administration of AE2 to mice significantly improves glucose intolerance and insulin resistance that are induced by human SeP administration. Furthermore, excess SeP administration significantly decreases pancreas insulin levels and high glucose-induced insulin secretion, which are improved by AE2 administration. Epitope mapping reveals that AE2 recognizes a region of human SeP adjacent to the first histidine-rich region (FHR). A polyclonal antibody against the mouse SeP FHR improves glucose intolerance and insulin secretion in a mouse model of diabetes. This report describes a novel molecular strategy for the development of type 2 diabetes therapeutics targeting SeP.

## Abstract (web summary)

Selenoprotein P is secreted by the liver and when present in excess it promotes development of type 2 diabetes. Here the authors develop neutralizing antibodies to target human and mouse selenoprotein P, and show that they improve insulin secretion and glucose tolerance in mouse models.

## Introduction

Selenoprotein P (SeP; encoded by *SELENOP*) is a heavily glycosylated extracellular protein produced mainly by the liver^1, 2^. SeP contains the essential trace element selenium (Se) as selenocysteine (Sec), which is encoded by UGA, formerly known as a stop codon in mRNA^3, 4^. Sec-containing proteins are termed selenoproteins, and the 'P' in SeP denotes its presence in plasma. SeP functions as an Se transporter to deliver Se from the liver to other tissues, playing an essential in vivo role to maintain appropriate Se levels in tissues^5–7^. Lipoprotein receptors such as apolipoprotein E receptor-2 (ApoER2) and megalin have been identified as receptors for SeP^8, 9^. Very recently, we have reported low-density lipoprotein (LDL) receptor-related protein 1 (LRP1) as a novel receptor for SeP^10^. SeP is considered to bind to cell surface receptor proteins and then to supply Se via endocytosis. Se is necessary for the synthesis of antioxidative selenoproteins, such as glutathione peroxidases (GPxs) and thioredoxin reductases (TrxRs)^4^. Thus, SeP plays an important role in the cellular antioxidative system via the maintenance of these selenoproteins. Furthermore, SeP possesses multifunctional properties such as GPx-like enzyme activity, peroxynitrite scavenging and metal binding activity^11–13^. SeP is composed of two domains: one possesses GPx-like enzyme activity containing one Sec residue in the N-terminal region and the other functions as an Se supplier containing nine Sec residues in the C-terminal region^14^. These domains are connected by a bridge containing two regions that are rich in histidine, which has the typical heparin-binding motif XBBXB (B is a basic amino acid)^3, 15^.

We have previously found that SeP serves as a hepatokine, a liver-derived secretory protein, causing glucose intolerance and insulin resistance in type 2 diabetes^16^. SeP is up-regulated in the liver of type 2 diabetes patients and in rodent models of the disease, such as KKAy mice and mice fed a high-fat, high-sucrose diet (HFHSD)^16^. Injection of human SeP corresponding with an incremental change of SeP serum levels found in patients with type 2 diabetes significantly induces glucose intolerance and insulin resistance in treated mice^16^. High levels of SeP impair insulin signalling and dysregulate glucose metabolism both in liver and muscle via the inactivation of adenosine monophosphate-activated protein kinase (AMPK)^16^. We have recently reported that deficiency of muscle LRP1 abolishes SeP-induced suppression of AMPK phosphorylation during exercise and enhances responsiveness to exercise training in mice^10^. Taken together, previous studies suggest that increased SeP is a specific therapeutic target for type 2 diabetes^16–18^; however, therapeutic agents targeting SeP have not yet been developed.

These lines of evidence suggest that inhibition of SeP binding and Se-supply activity would improve insulin resistance and glucose metabolism. Based on this hypothesis, we evaluate the effects of SeP blockade on glucose intolerance using SeP-neutralizing antibodies (Abs). We show that administration of SeP-neutralizing Ab could improve insulin secretion and glucose sensitivity in mouse models of type 2 diabetes. This report describes a novel molecular strategy for the development of type 2 diabetes therapeutics targeting SeP.

## Results

### Identification of neutralizing antibody for human SeP

**Fig. 1:**
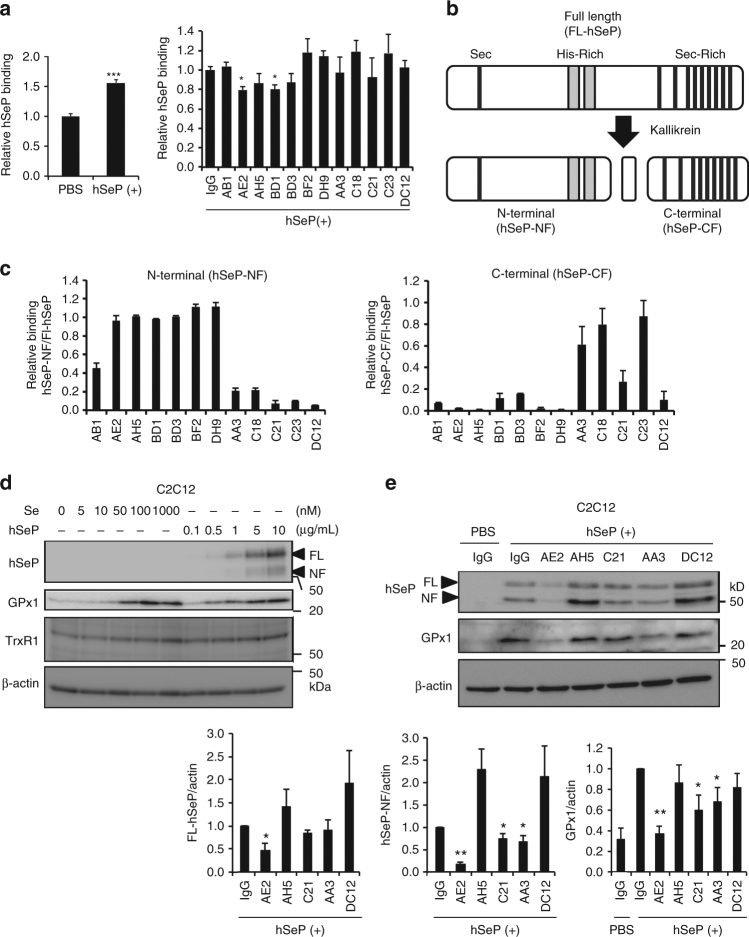
Identification of antibodies inhibiting the binding and selenium supply of SeP. **a** Inhibitory effects of monoclonal antibodies on the interaction between undifferentiated C2C12 cells and human SeP. C2C12 cells were incubated with purified human SeP (hSeP) protein (0.5 µg/mL) in the absence (left panel) or the presence (right panel) of each mAb (10 µg/mL) at 4 °C, and then SeP binding was analysed (*n* = 3, means ± s.d.). ***P < 0.001, Student's *t*-test (left panel), *P < 0.05, vs. control IgG, Tukey-ANOVA (right panel). **b** Schematic representation of limited proteolysis of human SeP by plasma kallikrein. The sequential limited proteolysis of full-length-hSeP (FL-hSeP) by plasma kallikrein (R235-Q236 and R242-D243) resulted in the generation of N-terminal (hSeP-NF) and C-terminal fragments (hSeP-CF). **c** Immunoreactivity of each mAb against hSeP-NF and hSeP-CF was determined by direct ELISA (*n* = 3, means ± s.d.). Relative binding, hSeP-NF immunoreactivity/FL-hSeP immunoreactivity and hSeP-CF immunoreactivity/FL-hSeP immunoreactivity, is shown. **d** Differentiated C2C12 myocytes were treated with each concentration of sodium selenite (Se) and hSeP for 24 h, as described in the Methods, and then whole-cell lysates were analysed by western blotting with anti-hSeP Ab BD1, anti-GPx1 Ab and anti-TrxR1 Ab KB12. **e** Effects of monoclonal antibodies for Se-supply activity of human SeP in C2C12 myocytes. C2C12 myocytes were treated with hSeP (0.5 µg/mL) in the presence of each mAb (10 µg/mL) for 24 h. Human SeP and GPx1 levels in whole-cell lysates were determined by western blotting (*n* = 3, means ± s.d.). The band intensity of hSeP was only evaluated in hSeP-treated cells. **P < 0.01, *P < 0.05, vs. control IgG of hSeP-treated cells, Tukey-ANOVA

To develop SeP-neutralizing Ab, we first investigated the cell surface binding of purified human SeP protein (hSeP) by using cultured cells. To avoid the effects of endogenous SeP, cells with low levels of expression of *SELENOP* were used (Supplementary Fig. 1a). In preliminary experiments using undifferentiated C2C12 cells, significant binding of hSeP was detected (Fig. 1a). We next incubated undifferentiated C2C12 cells with hSeP and each monoclonal antibody (mAb). We found that mAbs such as AE2 and BD1 significantly inhibited the binding of hSeP to C2C12 cells (Fig. 1a). We also observed significant binding of hSeP and inhibitory effects of several mAbs in Jurkat cells (Supplementary Figs. 1b and 1c).

The treatment of full-length hSeP (FL-hSeP) with plasma kallikrein results in sequence-limited proteolysis (Arg-235–Gln-236 and Arg-242–Asp-243)^14^, which generated N-terminal (hSeP-NF) and C-terminal fragments (hSeP-CF) (Fig. 1b). Binding-inhibitory Abs such as AE2, BD1, BF2 and DH9 had immunoreactivity with hSeP-NF, while AA3 had hSeP-CF (Fig. 1c). DC12 did not show distinct immunoreactivity against either fragment, suggesting that DC12 only recognizes intact hSeP (Fig. 1c).

To examine the effects of mAbs on the cellular uptake and supply of Se by hSeP, we incubated differentiated C2C12 myocytes with various concentrations of hSeP and sodium selenite. We found that the levels of cellular selenoproteins, such as GPx1 and TrxR1, increased in an hSeP- and sodium selenite-concentration-dependent manner (Fig. 1d), suggesting that these levels of selenoproteins, particular GPx1 levels, were indicators of Se-supply. We also found a concentration-dependent increase in hSeP levels in whole-cell lysates of hSeP-treated C2C12 myocytes (Fig. 1d). We have confirmed that in our experimental condition, LRP1 functions as an SeP receptor of C2C12 myocytes by using *LRP*1-siRNA (Supplementary Fig. 2a). We developed a similar system to analyse hSeP uptake and Se-supply using Jurkat cells (Supplementary Fig. 2b), and found that hSeP immunoreactivity was mainly inside of hSeP-treated cells (Supplementary Fig. 2c). These results suggest that the hSeP level in whole-cell lysates could be an indicator of hSeP uptake even though the precise contribution of hSeP-binding to the cell surface could not be evaluated by this method.

We next investigated the effects of mAb co-incubation on the cellular uptake and Se-supply of hSeP. Addition of several mAbs decreased the cellular levels of hSeP and GPx1 (Fig. 1e and Supplementary Fig. 2d). The strongest inhibitor of hSeP uptake and Se-supply was the anti-hSeP mAb clone AE2 (AE2). AE2 significantly inhibited the cellular uptake of hSeP in Jurkat cells (Supplementary Fig. 3a).

hSeP treatment results in a significant increase in insulin resistance of C2C12 myocytes^16^. We further evaluated the effects of hSeP on cellular redox status and the inhibitory effects of AE2 on insulin signal, and found that AE2 significantly suppressed the elevation of reduced glutathione (GSH) levels induced by hSeP (Supplementary Fig. 3b). We also found that AE2 treatment significantly increased Akt phosphorylation induced by insulin in hSeP-treated C2C12 myocytes, suggesting that AE2 significantly improved insulin resistance (Supplementary Fig. 3c). These results suggest that AE2 inhibits uptake and Se-supply activity of SeP, acting as a neutralizing Ab for hSeP in vitro.

### Effects of human SeP-neutralizing antibody in vivo

**Fig. 2:**
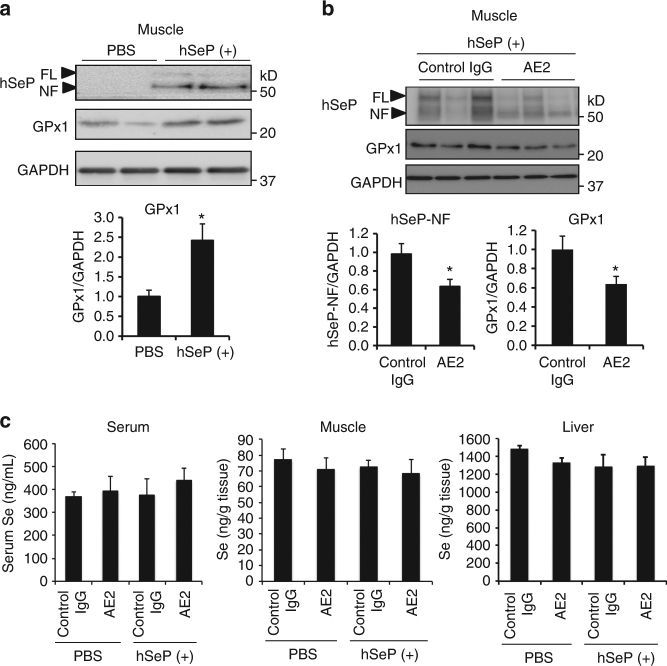
Effects of AE2 mAb on cellular uptake and Se-supply activity of SeP in vivo. **a** Following the scheme shown in Supplementary Fig. 4a, C57BL/6 J mice (female, 9 weeks old) were injected twice with purified hSeP (1 mg/kg intraperitoneally) or vehicle control (PBS intraperitoneally) 12 and 2 h before sampling. After perfusion with saline, excised skeletal muscle tissues were homogenized and protein samples were analysed by western blotting with anti-hSeP Ab BD1 and anti-GPx1 Ab (*n* = 3, means ± s.e.m.). **b**, **c** AE2 and control rat IgG1 (20 mg/kg intraperitoneally) were administered 2 h before the first injection of hSeP. Two hours after the second injection of hSeP, muscle tissues were homogenized and protein samples were analysed by western blotting (**b**, *n* = 11–13, means ± s.e.m.). *P < 0.05, Student's *t-*test (**a**, **b**). Se was assayed in serum and homogenate samples of muscle and liver, as described in the Methods (**c**, *n* = 5–7, means ± s.e.m.)

Next, we investigated the inhibitory activity of AE2 for hSeP in vivo. Female C57BL/6J mice were twice administered purified hSeP (1 mg/kg body weight) intraperitoneally (ip), 12 and 2 h before sampling times as described in Supplementary Fig. 4a, in which insulin signalling and glucose metabolism are impaired^16^. We determined the uptake of hSeP by several tissues in treated mice by western blotting using mAb specific for hSeP after perfusion of saline. We observed bands corresponding to FL-hSeP and hSeP-NF in the skeletal muscle of treated mice (Fig. 2a), and a significant increase of GPx1 levels in this condition (Fig. 2a). Next, we confirmed that the serum concentration of anti-hSeP Ab was sustained 24 h after either ip or intravenous administration (Supplementary Fig. 4b). A concentration-dependent study of AE2 using C2C12 myocytes suggested that a 50% inhibitory effect of AE2 was reached at around 2.5–5 µg/mL AE2, which is 5- to 10-fold volume (g/g) of hSeP (Supplementary Fig. 4c). Thus, AE2 (20 mg/kg body weight, 20-fold volume of hSeP) was administered 2 h before the first hSeP treatment. As shown in Fig. 2b, hSeP-NF and GPx1 levels significantly decreased after administration of AE2. These results suggest that AE2 inhibits uptake and Se-supply activity of SeP, acting as a neutralizing Ab for hSeP in vivo.

To evaluate the effects of hSeP- and AE2 treatment on Se homoeostasis, the Se content of serum, skeletal muscle, and liver was determined 2 h after the second administration of hSeP. The serum Se content in hSeP- and AE2-treated mice tended to increase; however, no significant difference between each group was observed in serum, skeletal muscle, and liver (Fig. 2c). To understand the change of Se metabolism in hSeP-treated mice, serum levels of administered hSeP and endogenous mouse SeP (mSeP) were further evaluated. We found hSeP-specific bands in the serum of hSeP-treated mice, while endogenous mSeP was decreased significantly (Supplementary Fig. 5a). These results suggest that the small difference in serum Se levels between PBS- and hSeP-treated mice might be caused by a decrease in the amount of endogenous mSeP.

### Effects of SeP-neutralizing Ab AE2 on glucose metabolism

**Fig. 3:**
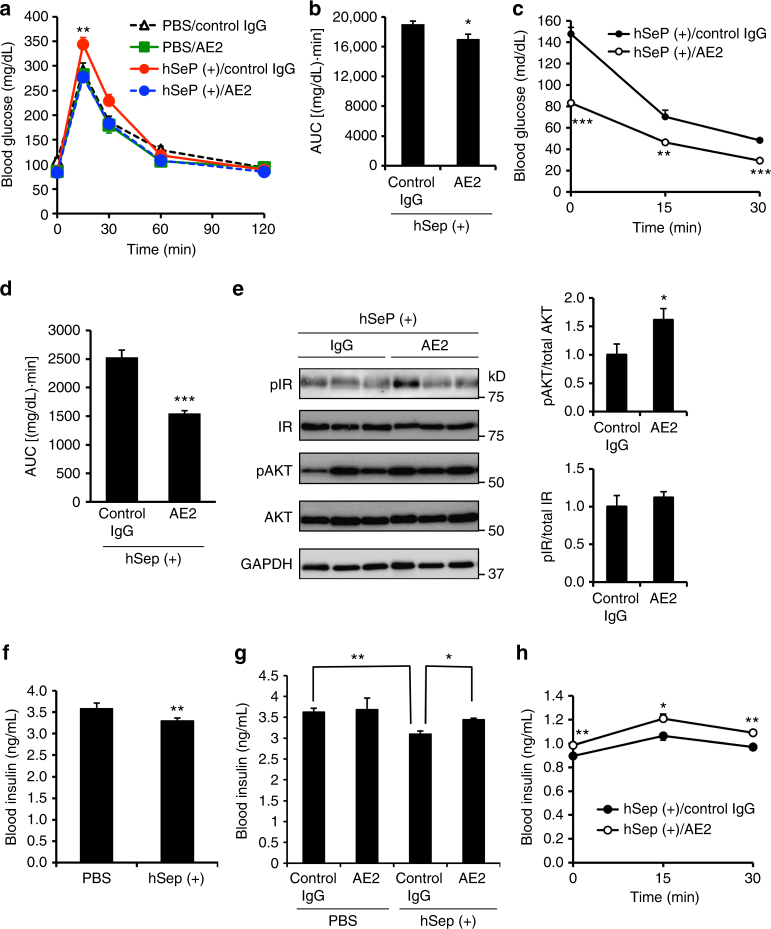
The SeP-neutralizing Ab improved glucose metabolism impaired by excess SeP. **a**, **b** Neutralizing mAb AE2 improved glucose tolerance impaired by human SeP administration in vivo. After the administration of AE2 and hSeP following the scheme of Supplementary Fig. 4a, glucose (1.5 g/kg) was administered, and then blood glucose was determined (**a**, *n* = 6–8, means ± s.e.m.). Open triangle, PBS/control IgG; green square, PBS/AE2; red circle, hSeP(+)/control IgG; blue circle, hSeP(+)/AE2. **P < 0.01, when compared hSeP(+)/control IgG group with other groups, Tukey-ANOVA. Area under the curve for blood glucose levels is shown in **b** (*n* = 6–8, means ± s.e.m.). *P < 0.05, Student's *t*-test. **c**–**e** Neutralizing mAb AE2 improved insulin resistance impaired by SeP administration in vivo. After the administration of AE2 and hSeP following the scheme shown in Supplementary Fig. 4a, insulin (0.5 U/kg) was administered, and blood glucose was determined (**c**, *n* = 5–8, means ± s.e.m.). **P < 0.01, ***P < 0.001, Tukey-ANOVA. Area under the curve for blood glucose levels is shown in **d** (*n* = 5–8, means ± s.e.m.). ***P < 0.001, Student's *t-*test. Effect of AE2 mAb administration on phosphorylation of insulin receptor (IR) and Akt in skeletal muscle (**e**). In AE2- and hSeP-treated mice, muscle tissues were excised 15 min after insulin administration (0.5 U/kg), and analysed by western blotting (*n* = 5–8, means ± s.e.m.). *P < 0.05, Student's *t*-test. **f** Blood insulin levels were significantly decreased by human SeP administration. Blood insulin levels were determined 2 h after second hSeP or PBS administration under feeding conditions (*n* = 5, means ± s.e.m.). **P < 0.01, Student's *t-*test. **g** Blood insulin levels were significantly increased by AE2 administration under feeding conditions (*n* = 5, means ± s.e.m.). *P < 0.05, **P < 0.01, Tukey-ANOVA. **h** Blood insulin levels were significantly increased during a glucose-tolerance test. At each designated time, blood insulin was determined (*n* = 7–8, means ± s.e.m.). *P < 0.05, **P < 0.01, Tukey-ANOVA (**c**, **h**). Closed circle, hSeP(+)/control IgG; open circle, hSeP(+)/AE2

We examined the effects of SeP neutralizing AE2 on glucose metabolism impaired by administration of hSeP in vivo. AE2 and purified hSeP were administered as described above, and then a glucose tolerance test was conducted. Treated animals were fasted 12 h before the glucose tolerance test (Supplementary Fig. 4a). Neither AE2 nor control IgG affected blood glucose levels in untreated control mice (Fig. 3a), while AE2 treatment of hSeP-treated mice significantly suppressed elevation of blood glucose (Fig. 3a, b), which suggests that AE2 improves glucose tolerance impaired by hSeP treatment.

We next conducted an insulin tolerance test to determine the effects of AE2 on insulin sensitivity. We fasted hSeP-treated mice 4 h before the insulin tolerance test (Supplementary Fig. 4a) and found that basal blood glucose levels markedly decreased in mice administered AE2 compared with hSeP-treated mice administered control IgG at time 0 (Fig. 3c). Administering AE2 significantly decreased blood glucose levels in hSeP-treated mice during the insulin tolerance test (Fig. 3c, d), while administering AE2 alone or control IgG to PBS-treated mice did not influence blood glucose levels so much (Supplementary Fig. 5b). Next, we evaluated the change in insulin signalling molecules such as phosphorylation of insulin receptor (IR) and Akt following the scheme of the insulin tolerance test. We found significant elevation of insulin-stimulated phosphorylated Akt in the skeletal muscle of AE2-treated mice, suggesting that AE2 administration improves insulin resistance impeded by hSeP treatment (Fig. 3e). The effects of AE2 injection on levels of hepatic lipid, such as total cholesterol and triglyceride, were evaluated; however, no significant difference was observed between groups (Supplementary Table 1).

Next, we determined the effects of AE2 on insulin secretion. We first examined the sole effect of hSeP treatment on blood insulin levels, and we found that in the absence of Ab administration, hSeP treatment resulted in a significant decrease in blood insulin of fed mice (Fig. 3f). Decreased insulin levels as a result of hSeP treatment were improved by AE2 administration in fed mice (Fig. 3g). Furthermore, in a glucose tolerance test, blood insulin levels were increased significantly by administering AE2 at time 0, and high levels of insulin were sustained (Fig. 3h). These results suggest that hSeP neutralizing Ab AE2 improves insulin levels impeded by hSeP treatment.

### Effects of excess SeP and its neutralizing Ab on pancreas

**Fig. 4:**
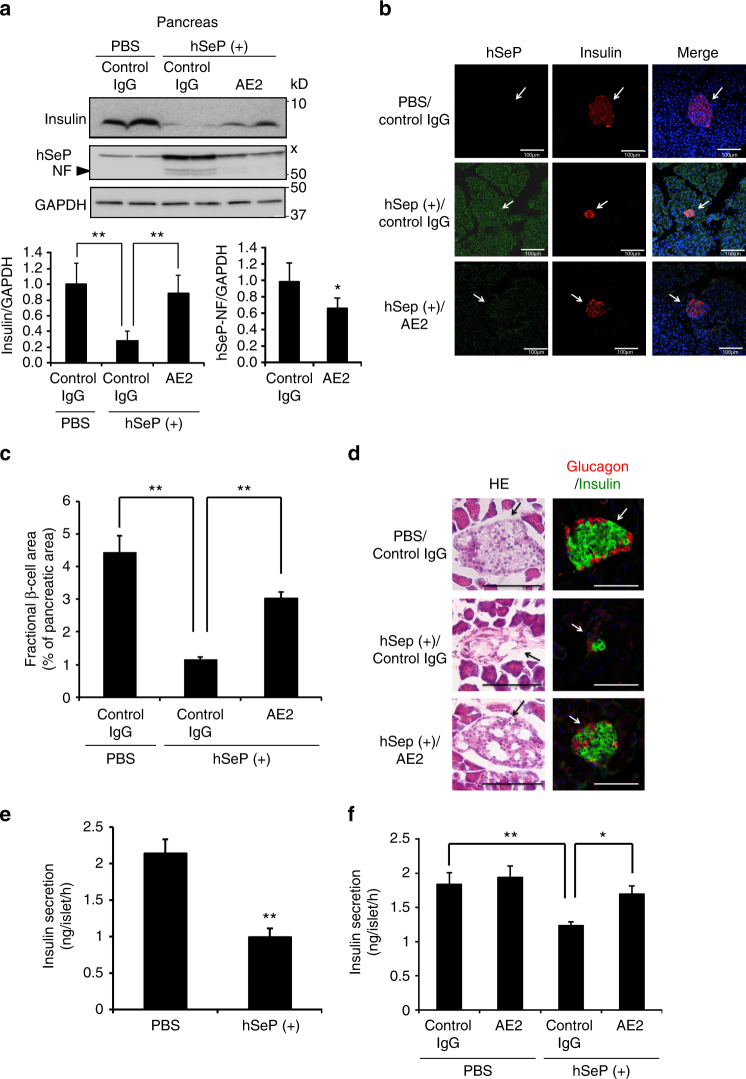
The SeP-neutralizing Ab improved insulin secretion impaired by SeP. **a** SeP-neutralizing mAb AE2 improved pancreas insulin levels. After administration of AE2 and hSeP, pancreas tissues were homogenized and analysed by western blotting (*n* = 5–8, means ± s.e.m.). Non-specific bands are indicated by an x. **P < 0.01, Tukey-ANOVA. *P < 0.05, Student's *t*-test. **b**, **c** Neutralizing mAb AE2 improved pancreatic β-cell area. Pancreas tissues from AE2- and hSeP-treated mice were examined immunohistochemically using anti-insulin Ab (indicative of β-cells) and anti-hSeP Ab **b**. Cell nuclei were stained with DAPI (blue). The distribution of hSeP in pancreatic β-cells was decreased in mice administered AE2. The proportion of β-cell area to total pancreatic area was determined (**c**, *n* = 3, means ± s.e.m.). **P < 0.01, Tukey-ANOVA. **d** Histochemical analysis of pancreas tissues from AE2- and hSeP-treated mice using haematoxylin and eosin stain (HE), anti-insulin Ab (green) and Glucagon (red, indicative of α-cells). Scale bars = 100 µm (**b**, **d**). **e** Insulin secretion of isolated rat islets was significantly decreased by excess human SeP. Isolated rat islets were incubated with 10 µg/mL hSeP for 24 h, and then insulin secretion induced by high glucose condition was evaluated, as described in the Methods (*n* = 3, means ± s.d.). **P < 0.01, Student's *t*-test. **f** Insulin secretion of isolated rat islets was significantly improved by AE2. Isolated rat islets were incubated with 10 µg/mL hSeP in the presence of AE2 mAb or control IgG (500 µg/mL) for 24 h, and then insulin secretion was evaluated (*n* = 3, means ± s.d.). *P < 0.05, **P < 0.01, Tukey-ANOVA

We further investigated the effects of AE2 on insulin and hSeP levels in the pancreas of hSeP-treated mice. As shown in Supplementary Fig. 5c, a band specifically corresponding to hSeP-NF was detected in the pancreas of hSeP-treated mice. A non-specific band was detected around the band of FL-hSeP, and the determination of FL-hSeP levels was difficult. We found a significant decrease in insulin levels in the pancreas of hSeP-treated mice (Supplementary Fig. 5c). By contrast, AE2 treatment significantly increased insulin levels in the pancreas of hSeP-treated mice (Fig. 4a). AE2 treatment also significantly decreased SeP-NF levels in the pancreas (Fig. 4a). Immunohistochemical analysis of pancreatic islets using anti-insulin Ab showed a reduction of the size of the islets in hSeP-treated mice and suggested the protective effects of AE2 administration (Fig. 4b; Supplementary Fig. 6a). Quantitative analysis of the islet area in each pancreas showed a significant decrease in the area of the islets in hSeP-treated mice and protective effects of AE2 administration (Fig. 4c). Further, we could immunohistochemically determine the distribution of hSeP in pancreatic islets, and its inhibitory effects of AE2 on the distribution (Fig. 4b). Collectively, these results suggest that excess hSeP induces a reduction of insulin levels in pancreatic β-cells and that AE2 prevent the effects of hSeP. Pancreatic islet morphology was further assessed by using haematoxylin and eosin (HE) staining and immunostaining with a marker for α-cells. The islets of hSeP-treated mice showed a disturbed morphology with irregular shape and a concomitant decrease of α-cells with β-cells (Fig. 4d). The effects of hSeP treatment were partly rescued by AE2 administration.

The effects of excess hSeP on pancreatic islets were further evaluated by isolating the islets. We found that treatment with excess hSeP for 24 h significantly decreased the insulin secretion induced by high levels of glucose (Fig. 4e). Furthermore, in hSeP-treated groups, AE2 treatment significantly increased insulin secretion by the isolated pancreatic islets (Fig. 4f). To explore the mechanism of islet disorder induced by excess SeP, an equimolar amount of Sec to hSeP was added to isolated pancreatic islets as selenocystine, and then we evaluated the effects on insulin secretion. We found that addition of excess selenocystine significantly decreased insulin secretion by isolated islets (Supplementary Fig. 6b), suggesting that Se-supply from excess SeP is related to the decrease in insulin secretion by islets.

### Effects of excess SeP and its neutralizing Ab on MIN6 cells

**Fig. 5:**
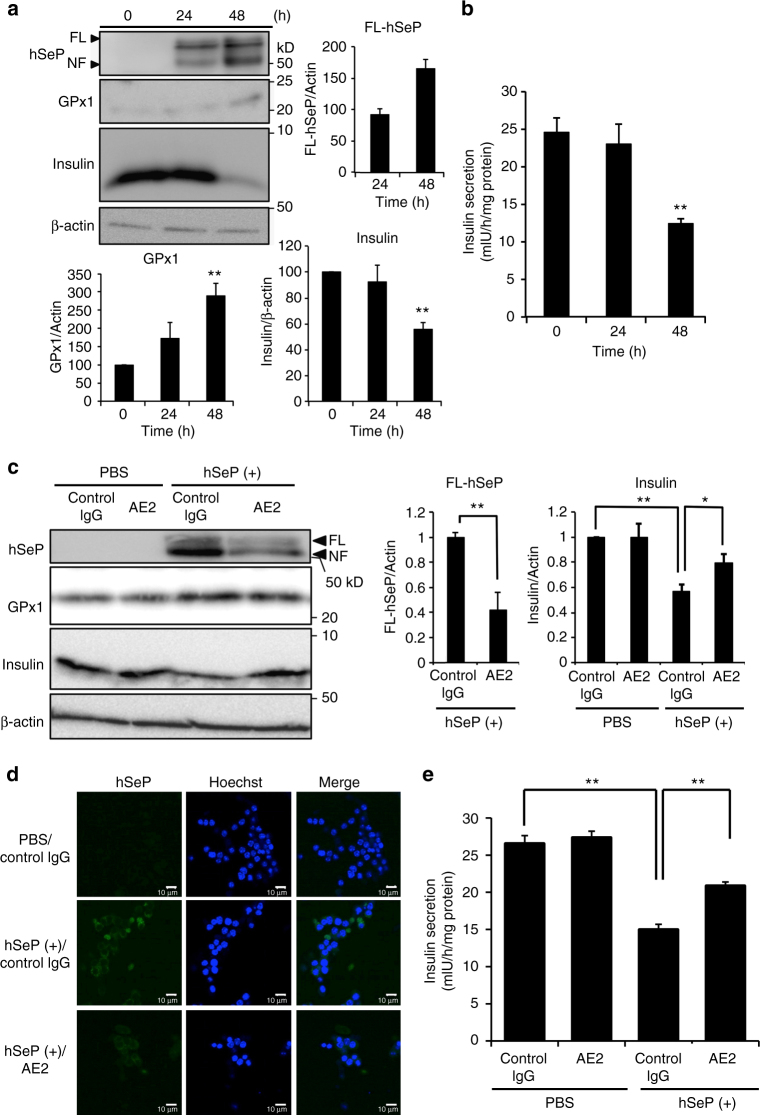
The SeP-neutralizing Ab improved insulin secretion by MIN6 cells. **a** Insulin levels of MIN6 cells were significantly decreased by excess human SeP. MIN6 cells were incubated with 10 µg/mL hSeP for the indicated time, and then whole-cell lysates were analysed by western blotting with anti-hSeP Ab BD1, anti-GPx1 Ab, and anti-insulin Ab (*n* = 3, means ± s.d.). The band intensity of hSeP was only evaluated in hSeP-treated cells for 24 and 48 h. **P < 0.01, vs. time 0, Tukey-ANOVA. **b** Insulin secretion by MIN6 cells was significantly decreased by excess human SeP. MIN6 cells were incubated with 10 µg/mL hSeP for the indicated time, and then insulin secretion induced by high glucose levels was evaluated (*n* = 3, means ± s.d.). **P < 0.01, vs. time 0, Tukey-ANOVA. **c**–**e** Insulin secretion of MIN6 cells was significantly improved by AE2. MIN6 cells were incubated with 10 µg/mL hSeP in the presence of AE2 and control IgG (500 µg/mL) for 48 h, and then whole-cell lysates were analysed by western blotting (**c**, *n* = 3, means ± s.d.). The band intensity of hSeP was only evaluated in hSeP-treated cells (**P < 0.01, Student's *t*-test). *P < 0.05, **P < 0.01, Tukey-ANOVA. MIN6 cells treated with hSeP and AE2 for 48 h were examined immunohistochemically using anti-hSeP Ab (**d**) or evaluation of insulin secretion induced by high glucose levels (**e**, *n* = 3, means ± s.d.). Scale bars = 10 µm. *P < 0.05, **P < 0.01, Tukey-ANOVA

To investigate the effects of SeP on insulin secretion by pancreatic β-cells, MIN6 cells, a model of β-cells, were used for further experiments. MIN6 cells were incubated with excess hSeP at 37 °C, and the cellular uptake of hSeP and supply of Se were determined by western blotting. We found that the levels of hSeP and cellular selenoprotein GPx1 increased in a time-dependent manner (Fig. 5a). We also found that cellular insulin levels of MIN6 cells were significantly decreased by excess hSeP for the 48 h treatment (Fig. 5a). Furthermore, excess hSeP treatment resulted in a decrease in insulin secretion induced by high levels of glucose (Fig. 5b). Selenocystine treatment of MIN6 cells also significantly decreased insulin secretion (Supplementary Fig. 6c). These findings suggest that supply of Se by excess hSeP was related to the decrease in insulin secretion.

The mRNA expression of SeP receptor genes such as *ApoER2, LRP1*, and *megalin* was observed in MIN6 cells, and the level of expression of *ApoER2* was higher than that of others (Supplementary Fig. 6d). Treatment of MIN6 cells with *ApoER2*-siRNA resulted in slight, but significant reduction of ApoER2 levels and hSeP levels, suggesting that ApoER2 plays, at least in part, a role for uptake of hSeP by MIN6 cells (Supplementary Fig. 7a). The addition of AE2 significantly decreased in the levels of cellular hSeP (Fig. 5c). AE2 addition also significantly suppressed the decrease of cellular insulin induced by excess hSeP (Fig. 5c). Inhibitory effects of AE2 on hSeP uptake in MIN6 cells were observed by immunohistochemical analysis (Fig. 5d). We found that AE2 addition significantly increased insulin secretion induced by high levels of glucose in MIN6 cells treated with excess hSeP (Fig. 5e).

### Epitope mapping and preparation of mouse SeP-neutralizing Ab

**Fig. 6:**
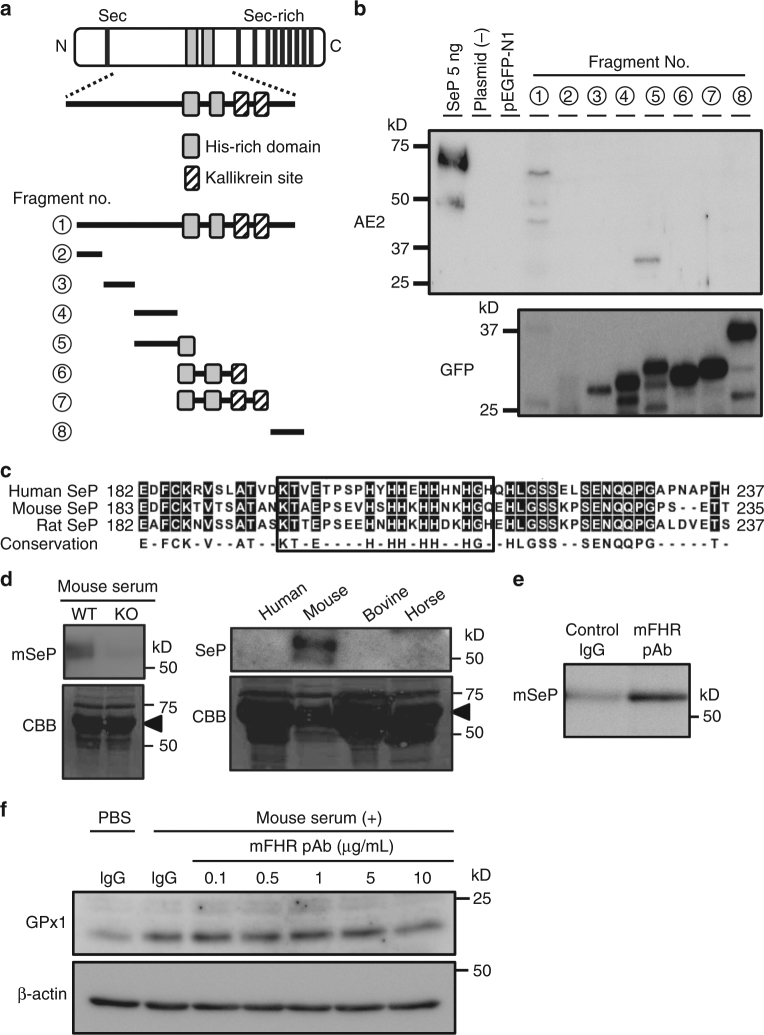
The identification of epitope recognized by SeP-neutralizing Abs. **a** Schematic outline of the epitope mapping approach. Green fluorescence protein (GFP)-tagged deletion constructs of hSeP were prepared. **b** Identification of AE2 mAb recognition site. Whole-cell lysates transfected GFP-tagged deletion constructs of hSeP were analysed by western blotting using anti-hSeP Ab AE2 and anti-GFP Ab. **c** Amino acid sequence homology around the first histidine-rich region (FHR) between three species. Predicted epitope of neutralizing antibody is enclosed. **d** Specificity and cross-reactivity of prepared polyclonal antibody for the FHR of mouse SeP (mFHR). Serum from wild type and SeP-KO mouse and other animal species was analysed by western blotting using mFHR pAb. As a loading control, separated proteins were stained with Coomassie Brilliant Blue R-250 (CBB). The major band derived from albumin is indicated. **e** Immunoprecipitation of mSeP by mFHR pAb. Mouse SeP in mouse serum was reacted with mFHR pAb- or control IgG-conjugated beads 4 °C for 2 h, and then immunoprecipitants were analysed by western blotting using mFHR pAb. **f** Evaluation of mFHR pAb against Se-supply activity by mSeP in C2C12 myocytes. Cells were treated with mouse serum (1%) in the presence of the indicated amount of mFHR pAb or control rabbit IgG (10 µg/mL) for 24 h. GPx1 levels were determined by western blotting

To identify the epitope of the neutralizing Abs, we transiently transfected HEK293 cells with green fluorescence protein-tagged hSeP deletion construct, and lysates of the cells were analysed by western blotting (Fig. 6a). Epitope mapping demonstrates that AE2 only recognizes a deletion construct (No. 5) containing sequences adjacent to the first histidine-rich region (FHR) (Fig. 6b). Another mAb, BD1, which inhibits binding, showed immunoreactivity with fragment Nos 5, 6, and 7, while non-inhibitory AH5 recognized fragment Nos 4 and 5 (Supplementary Fig. 7b). These results suggest that AE2 recognizes amino acid sequences, including those adjacent the FHR. The histidine-rich region of SeP has been identified as a heparin-binding site, and lipoprotein receptors play a role responsible for the cellular uptake of SeP^3, 15, 19^. Therefore, we investigated the effects of heparin and LDL on cellular uptake of hSeP by C2C12 myocytes. As shown in Supplementary Fig. 7c, addition of heparin (1 mg/mL) inhibited the cellular uptake of hSeP, as reported previously^19^. By contrast, addition of LDL (100 µg/mL) at 10% serum concentration did not show obvious inhibitory effects for cellular uptake of hSeP (0.5 µg/mL), equivalent to 10% serum concentration, by C2C12 myocytes (Supplementary Fig. 7c).

Sequence homology around the FHR between three species is shown in Fig. 6c. Because AE2 did not show any immunoreactivity against mSeP, we prepared neutralizing Abs for mSeP using the amino-acid sequence adjacent to the mouse FHR as an immunogen. Polyclonal Abs against mSeP FHR (mFHR pAb) reacted with mSeP specifically, and we observed disappearance of the mSeP band in the serum of SeP knockout mice (Fig. 6d). mFHR pAb recognized mSeP, but not SeP from other animal species (Fig. 6d). The immunoreactivity of mFHR pAb for undenatured mSeP was confirmed by immunoprecipitation of mSeP in mouse serum (Fig. 6e). The supply of Se by mSeP and inhibitory effects of mFHR pAb was studied using mouse serum and C2C12 myocytes based on the cellular protein levels of GPx1. Addition of mFHR pAb (10 µg/mL) decreased GPx1 levels, suggesting suppression of the supply of Se by mSeP (Fig. 6f).

### Effects of mouse SeP-neutralizing Ab on KKAy mice

**Fig. 7:**
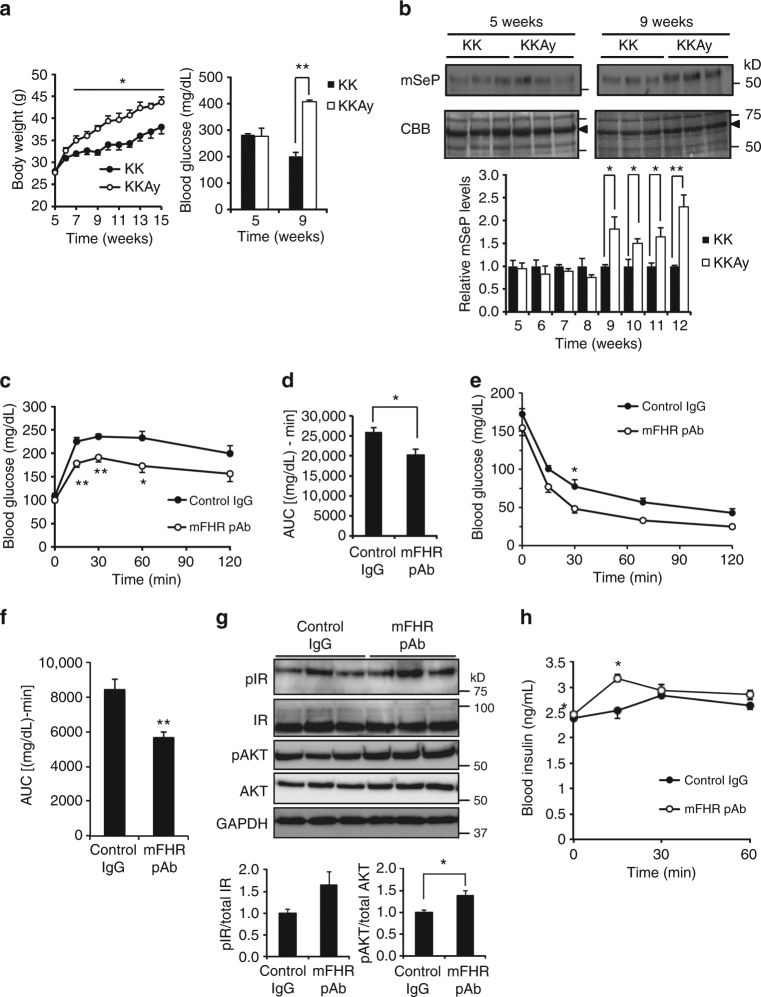
Improvement of glucose metabolism in the KKAy mouse by SeP-neutralizing Ab. **a**, **b** Time-dependent change of body weight, blood glucose (**a**) and plasma mSeP level (**b**) concentration in KKAy mice (*n* = 3, means ± s.e.m.). Closed circle, KK; open circle, KKAy. Graphs display the results of densitometric quantification, normalized to major protein (albumin, indicated by black arrowhead) stained with CBB (**b**, *n* = 3, means ± s.e.m.). **P < 0.01, *P < 0.05, Tukey-ANOVA (**a**, **b**). **c**, **d** Improvement of glucose tolerance in KKAy mice administered mSeP-neutralizing Ab. Mouse SeP neutralizing mFHR pAb or control rabbit IgG (25 mg/kg intraperitoneally) was administered 24 h before a glucose tolerance test following the scheme shown in Supplementary Fig. 8a. Glucose (0.3 g/kg body weight) was administered intraperitoneally, and blood glucose was determined at the indicated times (**c**, *n* = 5, means ± s.e.m.). *P < 0.05, **P < 0.01, Tukey-ANOVA. Area under the curve for blood glucose levels is shown in **d** (*n* = 5, means ± s.e.m.). *P < 0.05, Student's *t-*test. **e**–**g** Improvement of insulin resistance in KKAy mice injected with mSeP-neutralizing Ab. Mouse SeP neutralizing mFHR pAb was administered 48 h before an insulin-tolerance test following the scheme shown in Supplementary Fig. 8a. Insulin (10 U/kg) was administered intraperitoneally, and then blood glucose was determined at the indicated times (**e**, *n* = 5, means ± s.e.m.). *P < 0.05, Tukey-ANOVA. Area under the curve for blood glucose levels is shown (**f**, *n* = 5, means ± s.e.m.). **P < 0.01, Student's *t-*test. Phosphorylation of IR and Akt in skeletal muscle of mFHR pAb- or control IgG-treated KKAy mice was evaluated 15 min after insulin administration (**g**, *n* = 5, means ± s.e.m.). *P < 0.05, Student's *t-*test. **h** Improvement of insulin secretion in KKAy mice treated with mSeP-neutralizing Ab. Blood insulin levels were determined during the glucose-tolerance tests (*n* = 5, means ± s.e.m.). *P < 0.05, Tukey-ANOVA. Closed circle, control IgG; open circle, mFHR pAb (**c**, **e**, **h**)

We examined the effects of mSeP-neutralizing mFHR pAb on a mouse model of diabetes. Time-dependent change of mSeP, body weight and blood glucose concentration in KKAy mice was determined. The body weight of KKAy mice increased significantly compared with control KK mice from 7 weeks of age (Fig. 7a). A significant increase in blood glucose was observed at 9 weeks, but not at 5 weeks (Fig. 7a). Serum mSeP levels were significantly elevated in KKAy mice after 9 weeks (Fig. 7b). These observations indicate that in KKAy mice, mSeP levels increase with body weight and blood glucose.

Next, mFHR pAb was administered to KKAy mice at 9 weeks of age when serum mSeP levels were elevated (Fig. 7b), and then glucose- and insulin-tolerance tests were conducted, following the scheme shown in Supplementary Fig. 8a. Administration of mFHR pAb did not influence fasting blood glucose levels; however, mFHR pAb treatment significantly suppressed the elevation of blood glucose in the glucose tolerance test (Fig. 7c, d). In an insulin tolerance test, basal glucose levels tended to decrease in KKAy mice administrated mFHR pAb compared with control IgG at time 0, but the difference was not significant (Fig. 7e). Administering mFHR pAb significantly decreased blood glucose levels in KKAy mice during the insulin tolerance test (Fig. 7e, f). We found significant elevation of insulin-stimulated phosphorylation of Akt in the skeletal muscle of mFHR pAb-treated KKAy mice (Fig. 7g). Insulin-stimulated phosphorylation of IR also tended to increase (Fig. 7g). These results suggest that mFHR pAb improves insulin resistance of KKAy mice. We further investigated the effects of mFHR pAb on insulin secretion using a glucose-tolerance test. We found that mFHR pAb administration significantly elevated blood insulin levels induced by glucose administration (Fig. 7h). These findings suggest that in addition to the improvement of glucose intolerance and insulin resistance, mFHR pAb improves the insulin secretion in KKAy mice.

The effects of mFHR pAb on the metabolic phenotype of KKAy mice were further evaluated. The Se contents in serum, skeletal muscle, and liver of mFHR pAb-injected KKAy mice were not significantly changed (Supplementary Fig. 8b). By contrast, administration of mFHR pAb significantly decreased hepatic total cholesterol (Supplementary Table 2). Hepatic triglyceride also tended to decrease. These results suggest beneficial effects of mFHR pAb on hepatic lipid metabolism in KKAy mice.

To evaluate whether the effects of mSeP-neutralizing Ab are specific to the mouse model of diabetes, mFHR pAb were administered to C57BL/6J mouse controls, and then glucose- and insulin-tolerance tests were conducted. No significant effects of mFHR pAb on either glucose tolerance or insulin sensitivity were observed in the control mice (Supplementary Figs. 9a and b). These results suggest that under these experimental conditions, mSeP-neutralizing Ab does not affect glucose metabolism significantly in control C57BL/6 J mice.

### Effects of mouse SeP-neutralizing Ab on mice fed an HFHSD

**Fig. 8:**
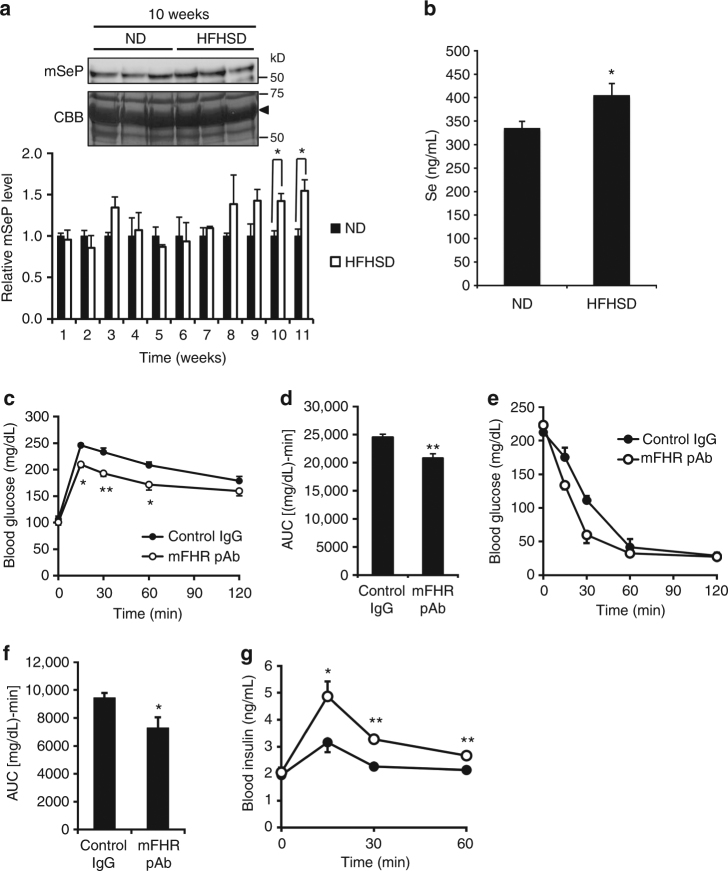
Improvement of glucose metabolism in HFHSD-fed mice by SeP-neutralizing Ab. **a** Time-dependent change of plasma mSeP level in mice fed a high-fat, high-sucrose diet (HFHSD) and a normal diet (ND). CBB staining was used as a control for protein loading. Graphs display the results of densitometric quantification, normalized to the major protein (albumin, indicated by black arrowhead) stained with CBB (*n* = 3, means ± s.e.m.). *P < 0.05, Tukey-ANOVA. **b** Elevation of plasma Se levels in mice fed an HFHSD. Plasma Se in mice fed an HFHSD and ND at 11 weeks was determined (*n* = 5, means ± s.e.m.). *P < 0.05, Student's *t*-test. **c**, **d** Improvement of glucose tolerance in mice fed an HFHSD treated with mSeP-neutralizing Ab. Mouse SeP-neutralizing mFHR pAb or control rabbit IgG (25 mg/kg ip) was administered 24 h before glucose-tolerance tests following the scheme shown in Supplementary Fig. 8a. Glucose (0.3 g/kg body weight) was administered intraperitoneally, and blood glucose was determined at the indicated times (**c**, *n* = 5, means ± s.e.m.). *P < 0.05, **P < 0.01, Tukey-ANOVA. Area under the curve for blood glucose levels is shown in **d** (*n* = 5, means ± s.e.m.). **P < 0.01, Student's *t*-test. **e**, **f** Improvement of insulin sensitivity in mice fed an HFHSD treated with mSeP-neutralizing Ab. Mouse SeP-neutralizing mFHR pAb was administered 48 h before insulin tolerance tests following the scheme shown in Supplementary Fig. 8a. Insulin (10 U/kg ip) was administered, and then blood glucose was determined at the indicated times (**e**, *n* = 5, means ± s.e.m.). The area under the curve for blood glucose levels is shown (**f**, *n* = 5, means ± s.e.m.). *P < 0.05, Student's *t-*test. **g** Improvement of insulin secretion in mice fed an HFHSD by mSeP-neutralizing Ab administration. Blood insulin levels were determined during glucose tolerance tests (*n* = 5, means ± s.e.m.). *P < 0.05, **P < 0.01, Tukey-ANOVA. Closed circle, control IgG; open circle, mFHR pAb (**c**, **e**, **g**)

We next examined the effects of mSeP-neutralizing mFHR pAb on mice fed with an HFHSD. Body weight significantly increased from 2 weeks after HFHSD feeding, blood glucose levels significantly increased from 3 weeks, and then mSeP levels significantly increased from 10 weeks (Fig. 8a; Supplementary Fig. 10a). At 11 weeks after HFHSD feeding, a significant increase in serum Se levels was found (Fig. 8b). Next, mFHR pAb was administered to the mice fed an HFHSD at 11 weeks after HFHSD feeding, and glucose- and insulin-tolerance tests were conducted (Supplementary Fig. 8a). Administration of mFHR pAb did not influence fasting blood glucose levels in mice fed an HFHSD, while mFHR pAb treatment significantly suppressed the elevation of blood glucose levels in the glucose-tolerance test (Fig. 8c, d). In the insulin-tolerance test, mFHR pAb tended to improve the sensitivity of mice fed an HFHSD to insulin (Fig. 8e) and significantly decreased the area under the curve of insulin-tolerance test was observed (Fig. 8f). We also investigated the effects of mFHR pAb on insulin secretion during the glucose-tolerance test, and we found that mFHR pAb administration significantly elevated blood insulin levels induced by glucose administration (Fig. 8g). These findings suggest that mFHR pAb improves glucose intolerance, insulin sensitivity, and insulin secretion in mice fed an HFHSD.

We determined hepatic lipid levels in mice fed an HFHSD. We found that hepatic total cholesterol and triglyceride levels tended to decrease, but no significant difference was observed (Supplementary Table 3). The change of Se levels in serum, skeletal muscle and liver was determined; however, no significant difference between mice administrated control Ab- or mFHR pAb were observed (Supplementary Fig. 10b).

## Discussion

In the present study, we have isolated SeP-neutralizing Abs that improve glucose intolerance, insulin resistance and insulin secretion in vitro and in vivo. We determined the site on SeP that might be important for its interaction with cells and tissues, suggesting a specific molecular target for therapeutic agents to treat type 2 diabetes.

Cellular GPx1 level is a suitable marker of cellular Se status^20^. We found that in the concentration-dependent study of sodium selenite and hSeP in C2C12 myocytes and Jurkat cells (Fig. 1d; Supplementary Fig. 2b), GPx1 levels reached a maximum around 100 nM sodium selenite in both cells, while maximal GPx1 levels were achieved by 5 µg/mL hSeP in C2C12 myocytes and 50 ng/mL in Jurkat cells, respectively. In addition, the levels of GPx1 in hSeP- and AE2-treated cells suggest that the efficacy of AE2 might be higher in C2C12 myocytes than it is in Jurkat cells (Fig. 1e and Supplementary Fig. 3a). Collectively, these results suggest the possibility that the efficacy of Se-supply of SeP and SeP-neutralizing Ab differs depending on the type of cells. Immunoreactivity for hSeP was detected mainly inside of hSeP-treated cells, and immunohistochemistry and western blotting of whole-cell lysates indicated that AE2 inhibits cellular uptake of hSeP. Because differenciated C2C12 myocytes are easily detached by incubation at 4 °C for a few hours, which might be a characteristic of these cell types, immunohistochemistry and binding assays using C2C12 myocytes were not successful. However, in western blotting of whole-cell lysates of C2C12 myocytes and binding assays using undifferentiated C2C12 cells, we found that AE2 could inhibit binding and incorporation of hSeP. Taken together, these experimental results in vitro strongly suggest that AE2 inhibits binding, cellular uptake and Se-supply by hSeP.

Se levels in serum and skeletal muscle of hSeP- and AE2-treated mice were not significantly changed even though selenoprotein levels in these biological samples were changed significantly (Fig. 2). The decrease in endogenous mSeP by hSeP treatment is considered a reason for the small difference in serum Se levels, suggesting an increase in mSeP excretion or a decrease of mSeP expression in liver, or both. Se content of standard laboratory mouse food in the present study was 0.4 mg Se/kg diet, which is higher than the established dietary requirement by mice of 0.1 mg Se/kg diet^21^. The Se content of HFHSD was 0.2 mg Se/kg diet. It appears that a high basal Se might also be a reason for non-significant changes of Se levels in biological samples. These results imply that in this experimental condition, unfavourable effects of excess SeP on glucose metabolism might be mediated by a part of selenoproteins, which contribute a minor portion of total Se.

*hSeP*-transgenic (TG) mice models, such as *hSeP*-TG *mSeP* ^−/−^ mice and WT mice treated with a hepatic overexpression plasmid encoding *hSeP*, have been established^17, 22^; however, changes in glucose metabolism in these TG mice have not been investigated in detail. We have already established experimental conditions under which injection of purified hSeP protein resulted in impairment of glucose and insulin tolerances^16^. Therefore, we employed hSeP-injection experiments in the present study. Our results demonstrate that hSeP treatment lowered insulin levels in both the blood and the pancreas, and that AE2 administration ameliorated hSeP-mediated hyperglycaemia and decrease in insulin levels. Although the sequence and interrelationship of biological events in the pancreas and skeletal muscle remain to be elucidated, SeP may disrupt glucose homoeostasis, not only by increasing insulin resistance, but also by impairing insulin secretion. To our knowledge, this study provides the first evidence suggesting that increased SeP impairs cellular insulin levels and insulin secretion in pancreatic β-cells. The protective effects of SeP-neutralizing Abs and hindering effects of excess selenocystine suggest a role for Se-supply in the failure of pancreas function by excess SeP. Pancreatic β-cells continuously synthesize and secrete insulin, which requires strict disulfide bond formation^23^. This task would make β-cells vulnerable to disorders of the redox environment, particularly protein folding in the endoplasmic reticulum, which could be induced not only by oxidative stress but also by an excessively reducing environment, such as found under high concentrations of dithiothreitol^23–25^. The present study suggests that SeP-neutralizing Ab could suppress the elevation of both GPx1 and reduced GSH levels, namely, the acceleration of a cellular redox environment, induced by SeP. It is likely that excessively reducing conditions relate to the inhibited insulin secretion caused by excess SeP. Several isoforms of SeP, such as N- and C-terminal fragments and truncated forms of SeP, have been reported^1, 14, 26^, and we have previously reported that Se content of purified FL-hSeP protein is 6.3, which is lower than that of theoretical value 10^27^. Nine Sec in the C-terminal part of SeP plays a major role in the supply of Se^14^, and it appears that an SeP isoform containing C-terminal Sec, such as FL-SeP or C-terminal fragment, might importantly influence the function of pancreatic β-cells. Mice expressing the SeP-fragment have been established^28, 29^; however, glucose metabolism in these mice has not been investigated in detail. In addition, details of SeP fragments in diabetes patients have not been fully elucidated, which need to be addressed in future studies.

In the islets of hSeP-treated mice, we found that the volume of both α- and β-cells decreased. Recent autopsy studies suggest that β-cell volume is reduced in patients with type 2 diabetes^30^, whereas alteration in α-cell volume remains controversial. The α-cell volume is reported to be reduced^31^ or unchanged with increased ratio to β-cell volume^32–34^ in type 2 diabetic islets. Our findings in the present study are in concert with our previous observation in patients with type 2 diabetes that glucagon secretion concomitantly decreases with insulin secretion^35^. It is interesting to note that abnormal cellular arrangement of α-cells in the core of the islets was clearly observed in the hSeP-treated islets partially rescued by AE2, whereas β-cells in the core of islets were surrounded by α-cells in the control mice (Fig. 4d). These findings suggest that hSeP treatment not only reduces the volume of both β- and α-cells, but also disturbs islet structures consisting of β- and α-cells. It is known that the arrangement of α- and β-cells in the islets is disturbed in diabetes model mice such as db/db mice^36^. One possible mechanism underlying such islet disarrangement might be transdifferentiation from β-cells to α-cells^37^, which should be a significant issue that needs to be elucidated in the future by testing the effects of excess SeP on cell differentiation and fate by using lineage tracing experiments.

Whether elevation of circulating SeP in type 2 diabetes is a cause or an effect of hyperglycaemia is controversial^38^. We and other groups found a positive correlation between blood concentrations of SeP and hyperglycaemia in people with type 2 diabetes^16, 38, 39^. These data raise the possibility that elevation of SeP is only an effect of hyperglycaemia in type 2 diabetes. However, the current data reveal that SeP-neutralizing Ab ameliorates insulin secretion and hyperglycaemia in rodent models of type 2 diabetes. Furthermore, genetic deletion or RNAi-mediated knockdown of SeP improves insulin resistance and hyperglycaemia in mice with obesity and type 2 diabetes^16^. Collectively, these data strongly suggest that overproduction of SeP functions as a causal factor for hyperglycaemia in type 2 diabetes by inducing both insulin resistance and impaired insulin secretion, at least in rodents. Overproduction of SeP and hyperglycaemia might contribute to a vicious positive feedback cycle that leads to the onset of more severe hyperglycaemia in type 2 diabetes. Prospective clinical studies are needed to determine whether high concentrations of blood SeP predict the future onset of type 2 diabetes in humans.

Several meta-analysis and a Cochrane review have questioned the basic paradigm between excess Se with risk for type 2 diabetes^40^, and baseline Se levels are thought to be a reason for the discrepancies observed in the human trials, which are dependent on several kinds of factors including food intake^41, 42^. We previously found that *SELENOP* expression is regulated via an AMPK–FoxOs axis, and metformin, a drug widely used for the treatment of type 2 diabetes, suppresses SeP expression via an AMPK–FoxO3a pathway^18, 43, 44^. Because conditions of high glucose and high lipid culture inhibit AMPK activity, these nutritional factors are thought to up-regulate SeP expression in hepatocytes. Production of SeP by hepatocytes plays a central role in Se homoeostasis and distribution of Se from the liver to peripheral tissues^45, 46^. As with other Sec-containing proteins, the synthesis of SeP is greatly influenced by Sec incorporation, which is significantly affected by Se status, the availability of sec-tRNA^[ser]sec^ and post-transcriptional factors^45–47^. Dietary Se incorporated into the liver is converted to sec-tRNA^[ser]sec^ or excretory metabolites such as selenosugars, and the syntheses of these seleno-metabolites compete with each other^48^. sec-tRNA^[ser]sec^ in the liver is used for the synthesis of cellular selenoproteins or SeP. Therefore, it is considered that adequate Se supply to maintain sec-tRNA^[ser]sec^ levels alone does not fully explain the high levels of serum SeP protein seen in mouse models of diabetic and in patients with type 2 diabetes. These results and previous reports suggest that both sufficient Se and an increase of SeP mRNA levels might be the key to the increase in serum SeP levels observed in diabetes.

The results of the present study showed clear changes in the levels of endogenous mSeP in the presence of exogenous hSeP (Supplementary Fig. 5a), and this phenomenon has also been suggested in a previous study^17^. These observations lead us to speculate that injection with hSeP reduced production of endogenous mSeP in the liver and/or increased excretion of mSeP into urine in mice. These speculations suggest that an unknown sensor-like system of SeP might be present to maintain whole-body Se homoeostasis, and that SeP receptors might be related to this system. The elucidation of this sensor-like system of SeP might be noteworthy for understanding not only the physiological control of SeP levels in blood but also the pathological change of SeP levels including type 2 diabetes.

The epitope of neutralizing Abs indicates the site in SeP that is important for the interaction with the cell surface^27, 49^. Heparin-binding properties of SeP have been known to mediate endocytosis of SeP, and lipoprotein receptors such as ApoER2, megalin and LRP1 have been identified as receptors for SeP^8–10, 15, 50^. The YWTD β-propeller domain of ApoER2 has been identified as an SeP-binding site, while the C-terminal domain of SeP alone can bind to ApoER2^51^. Furthermore, FL-SeP and its SeP-CF, but not its SeP-NF, can supply Se to cells^7, 14^. Results in the present study suggest that His-rich regions, particularly the first consecutive-His, should be molecular targets for inhibiting SeP binding and supply of Se. Addition of heparin inhibited the cellular uptake of hSeP in C2C12 myocytes, while we found indistinct effects of LDL addition on hSeP uptake in C2C12 myocytes (Supplementary Fig. 7c). Both LRP1 and LDLR are expressed in C2C12 myocytes^10^. The indistinct effects of LDL addition might be caused by the expression of multiple lipoprotein receptors. By contrast, Abs recognizing SeP-CF, such as AA3, also inhibit Se-supply of hSeP (Fig. 1e). Although we could not identify the epitope, the C-terminal domain of SeP is thought to be another molecular target to inhibit Se-supply activity. Reelin, a ligand of ApoER2, binds to the very-low-density-lipoprotein receptor, and the expression of these receptors changes the affinity of reelin to the cell surface, and subsequent signal transduction^52^. In accordance with previous reports^7, 10, 16, 51, 53^, we observed that the efficiency of supply of Se by SeP differs considerably between different cell types (Fig. 1d and Supplementary Fig. 2b). Further characterization of mediators of interactions between SeP and cells may provide further insight into the action of SeP.

mSeP-neutralizing pAb improved glucose tolerance in KKAy and HFHSD-fed mice, but not in control C57BL/6J mice. There are several speculations about these results. First, because control C57BL/6J mice showed normal glucose tolerance, mSeP-neutralizing Ab might not further reduce blood levels of glucose to the hypoglycaemia range by the action of counter-regulatory hormones of insulin, such as glucagon, adrenaline and cortisol^54^, in the normal mice. Second, because the concentration of mSeP in the blood has been reported to be 5.5-fold higher than that of hSeP^55^, the action of mSeP-neutralizing pAb might not be sufficient to neutralize endogenous mSeP and to induce hypoglycemia in the control mice. On the other hand, we have previously reported that treatment with hSeP in serum levels of 0.5–1.5 µg/mL, which correspond to the incremental changes of SeP in people with normal glucose tolerance to those with type 2 diabetes, causes hyperglycema in control C57BL/6 J mice^16^. Thus, we speculate that mSeP-neutralizing pAb attenuated hyperglycaemia in KKAy and HFHSD-fed mice only by neutralizing the incremental changes of blood SeP in type 2 diabetic mice from those in normoglycemic mice. Further experiments using mSeP-neutralizing Ab in a dose-dependent manner are needed to prove these speculations.

SeP-neutralizing Ab improved intrahepatic levels of total cholesterol and triglycerides in KKAy mice, suggesting a therapeutic benefit of SeP-neutralizing Ab for fatty liver disease as well as type 2 diabetes. However, injection with hSeP did not alter hepatic lipid levels directly in C57BL6/J mice (Supplementary Table 1). It is presumed that SeP-neutralizing antibody might decrease hepatic lipid contents by suppressing lipids efflux from the adipose tissue to the liver in KKAy mice; however, it is still unclear whether SeP acts on the adipose tissue. Additional studies using cultured adipocytes treated with hSeP will provide insights into the actions of SeP in the adipose tissue.

In hSeP- and AE2-treated mice, a change of basal blood glucose levels was observed in fasting procedure of an insulin-tolerance test (Fig. 3c). hSeP treatment induced the increase of insulin resistance and the decrease of insulin secretion, while AE2 administration improved these unfavourable effects. Thus, we consider that the effects of hSeP- and AE2 administration on insulin resistance and insulin secretion, at least in part, might be related to differences in basal blood glucose in this experimental model. By contrast, we found positive effects of mSeP-neutralizing Ab, mFHR pAb, on insulin resistance and insulin secretion in KKAy mice; however, a significant change of basal glucose levels during fasting procedure was not observed (Fig. 7). Therefore, we suggest that acute treatment with hSeP might be a reason for the change of basal glucose levels seen in hSeP-treated mice. Because AE2 administration lowered baseline glucose levels in the insulin-tolerance test, effects of AE2 on insulin resistance in vivo cannot be adequately evaluated in the hSeP-treated mouse model, although insulin-induced Akt phosphorylation in skeletal muscle was significantly increased by AE2 administration. Therefore, we tested the effects of mSeP-neutralizing Ab on glucose intolerance and insulin resistance in KKAy mice. Our findings suggested that increased mSeP chronically impairs glucose metabolism, insulin resistance and insulin secretion in KKAy mice, and that mSeP-neutralizing Ab ameliorate hyperglycaemia in KKAy mice, at least in part, by improving insulin resistance and insulin secretion. Excess hSeP treatment of C2C12 myocytes results in a decrease in glucose uptake^16^, and we consider that skeletal muscle, at least in part, enhances clearance of blood glucose in mice administered with SeP-neutralizing Ab. Furthermore, the positive effects of mSeP-neutralizing Ab administration in mice fed an HFHSD strongly suggest the effectiveness of this strategy. These findings suggest that suppression of increased SeP is a promising strategy for the therapeutic treatment of type 2 diabetes. To our knowledge, we also present the first evidence that the critical epitope of SeP targeted by effective Abs is conserved between humans and mice. This finding may provide a useful strategy in development of therapeutic antibody-based treatments targeting SeP.

In conclusion, the present study showed that administration of SeP-neutralizing Ab could improve, at least in KKAy mice model, glucose metabolism, insulin resistance and insulin secretion in vivo, and suggested a strategy of targeting SeP for treatment of type 2 diabetes. This report further provides evidence indicating that increased levels of SeP is a potent target that may allow therapies for type 2 diabetes to be developed.

## Methods

### Reagents

Dulbecco’s modified Eagle’s medium (DMEM), penicillin and streptomycin, and horse serum (HS) were obtained from Invitrogen (Thermo Fisher Scientific, Carlsbad, CA, USA). RPMI-1640 medium, glucose solution and 3,3′,5,5′-tetramethylbenzidine (TMB) were purchased from Merck (Darmstadt, Germany). Insulin was obtained from Eli Lilly (Indianapolis, IN, USA). Fetal bovine serum (FBS; Hyclone) and control rabbit IgG (02-6102) were purchased from Thermo Fisher Scientific (Logan, UT, USA). Control rat IgG1 mAb (MAB005) was obtained from R&D systems (Minneapolis, MN, USA). Human LDL (BT-903) was purchased from Alfa Aesar (Lancashire, UK). Heparin sodium (085-00134) was purchased from WAKO (Tokyo, Japan). Vitamin E isoform, α-tocopherol, was kindly provided by Eisai (Tokyo, Japan). Human SeP was purified from human plasma^27^. Human plasma was mixed with polyethylene glycol, and then proteins in the supernatant were separated by heparin-Sepharose CL-6B column, Q-Sepharose Fast Flow and Ni-NTA-agarose, respectively. Buffer of hSeP was changed by using PD-10 gel filtration column equilibrated with the desired buffer. Fragments of hSeP were prepared by using purified hSeP and human plasma kallikrein^14^. Purified hSeP was treated with plasma kallikrein, and then hSeP-NF and -CF were separated by Ni-NTA-agarose column chromatography. Human frozen plasma was kindly provided from Japanese Red Cross Kinki Block Blood Center (No. 25J0012). All other chemicals used were of the highest quality commercially available.

### Cell culture

MIN6 cells were originally from Miyazaki et al.^56^. All other cell lines used were obtained from the American Type Culture Collection (Manassas VA, USA). Mycoplasma contamination was not tested in the cells. MIN6 cells were routinely maintained in DMEM containing 10% heat-inactivated FBS, antibiotics (100 U/mL penicillin, 100 µg/mL streptomycin), 70 µM 2-mercaptoethanol and 40 mM sodium bicarbonate. Mouse myoblast C2C12 cells, Chinese hamster ovary (CHO) cells, human neuroblastoma SH-SY5Y cells and human glioma HTB14 cells were routinely maintained in DMEM containing 10% heat-inactivated FBS and antibiotics. Human T-cell lymphoma Jurkat cells were maintained in RPMI-1640 medium containing 10% heat-inactivated FBS and antibiotics. All cell lines were cultured at 37 °C under an atmosphere of 95% air and 5% CO_2_. To induce myogenic differentiation, the C2C12 cells were subsequently maintained in DMEM containing 0.5% HS and antibiotics.

### Real-time PCR analysis

Total RNA was extracted from each cultured cell using Tripure isolation reagent (Roche), and then reverse-transcribed using a PrimeScript RT reagent kit (TaKaRa Bio, Kyoto, Japan). Quantitative real-time PCR was performed using Power SYBR Green PCR Master Mix (Invitrogen) with the 7900HT Fast Real Time PCR System (Applied Biosystems) according to the manufacturer’s instructions. The housekeeping gene of ribosomal protein L32 (RPL32) was used as an endogenous control. The primers for amplification were as follows: *human SELENOP*, 5′-TGT GGA GCT GCC AGA GTA AAG-3′ (forward), 5′-CCA CAT TGC TGG GGT TGT CCT AT-3′ (reverse); *human RPL32*, 5′-CCC CTT GTG AAG CCC AAG A-3′ (forward), 5′-TGA CTG GTG CCG GAT GAAC-3′ (reverse); *mouse SELENOP*, 5′-ACT CGT CAA AAG TCG TCC GT-3′ (forward), 5′-ACC ACT GTC ACT TTG CCC TC-3′ (reverse); *mouse RPL32*, 5′-GAA ACT GGC GGA AAC CCA-3′ (forward), 5′-GGA TCT GGC CCT TGA ACC TTC-3′ (reverse); *mouse ApoER2*, 5′-CCT TGG TGT GGA GAT GCG AT-3′ (forward), 5′-ACA CTC TTC ACT GGA GCA CG-3′(reverse); *mouse LRP1*, 5′-CAC AAC CTC AAC GTC ATC CTG-3′ (forward), 5′-AGC ACA TTG TAC TCC TGG ATC-3′ (reverse); *mouse megalin*, 5′-TGC CTA AAG GGT TAC CCA CG-3′ (forward), 5′-TTG CTG GAT TTT GTC CTG GAG-3′ (reverse); *mouse LDLR*, 5′-CAT CCT CGG ACA TCC ACC C-3′ (forward), 5′-TTC GGT CGT GGC ACA AGA AC-3′ (reverse); *mouse VLDLR*, 5′-GAG TCT GAC TTC GTG TGC AAA-3′ (forward), 5′- GAA CCG TCT TCG CAA TCA GGA-3′ (reverse).

### Selenoprotein P binding assay

Undifferentiated C2C12 cells were incubated with 0.5 µg/mL purified hSeP in the presence of 10 µg/mL each anti-hSeP mAb for 1 h at 4 °C in DMEM containing 0.1% bovine serum albumin (BSA), 0.1% NaN_3_ and 50 mM HEPES (pH 7.4). Subsequently, treated cells were washed three times with ice-cold PBS (+), then incubated with 15 μg/mL rabbit anti-hSeP pAb for 1 h at 4 °C, and then fluorescein-conjugated anti-rabbit IgG (H+L) (Jackson Immuno Research, West Grove, PA, USA) for 1 h at 4 °C. The fluorescence was determined using a FLUOstar Galaxy microplate reader (BMG Labtech, Offenburg, Germany).

In the case of Jurkat cells, cells were cultured in the Se- and serum-free RPMI medium containing 2 µM α-tocopherol, 2.5 mg/mL BSA, 5 μg/mL human insulin, 5 μg/mL human transferrin and 92 nM FeCl_3_ for 24 h^57^. The cells at a density of 3 × 10^6^ cells/mL were further incubated with 5 μg/mL hSeP in the presence of 15 μg/mL each anti-SeP mAb for 1 h at 4 °C in a total volume of 1 mL of phosphate-buffered saline (PBS) containing 0.1% BSA and 0.1% NaN_3_. After washing, the cells were subsequently incubated with 15 μg/mL anti-hSeP pAb for 1 h at 4 °C, and then fluorescein-conjugated anti-rabbit IgG (H+L) (Jackson Immuno Research, West Grove, PA, USA) for 1 h at 4 °C. The fluorescence was analysed using a BD FACSAria IITM cell sorter (BD Bioscience, Franklin Lakes, NJ, USA). Data were collected from at least 10,000 events.

### Direct enzyme-linked immunosorbent assay (ELISA)

Ninety-six-well microtitre plates were coated with each hSeP proteins (200 ng/mL), such as FL-hSeP, hSeP-NF and hSeP-CF, in 50 mM sodium bicarbonate buffer (pH 9.6) for 1 h at room temperature. The wells were washed three times with wash buffer (PBS containing 0.05% Tween 20), and incubated at 37 °C with Blocking buffer (PBS containing 0.1% BSA) for 1 h. After washing, each anti-hSeP mAb (100 ng/mL in PBS containing 0.05% Tween 20 and 0.1% BSA) was added, and incubated at 37 °C for 1 h. After washing, horseradish peroxidase-conjugated anti-Rat IgG (H+L) or anti-mouse IgG (H+L) (Jackson Immuno Research) (400 ng/mL in PBS containing 0.05% Tween 20 and 0.1% BSA) was added, and incubated at 37 °C for 1 h. Finally, the plates were washed, and TMB was added. The enzyme-substrate reaction was allowed to proceed for 30 min. The reactions were stopped by the addition of 1 M H_2_SO_4_ to each well. The absorbance was read at 450 nm on an OPTImax plate reader (Molecular Devices, Sunnyvale, CA, USA).

### Western blotting

To obtain whole-cell lysates, treated cells were suspended in lysis buffer (50 mM Tris-HCl pH 7.5, 150 mM NaCl, 1% NP40, 0.1% SDS, 1% sodium deoxycholic acid with a cocktail of protease inhibitor (Nacalai Tesque, Kyoto, Japan) and phosphatase inhibitor (PhosSTOP, Roche, Mannheim, Germany) at 4 °C for 30 min. Nuclei and unlysed cellular debris were removed by centrifugation at 15,000 × *g* for 5 min. The protein concentration was determined by using a bicinchoninic acid protein assay kit (Pierce Biotechnology, Rockford, IL, USA) with BSA as the standard. The protein samples were separated by sodium dodecyl sulfate-polyacrylamide gel electrophoresis (SDS-PAGE) and subjected to western blotting with appropriate antibodies. In the case of western blotting of insulin, Tricine SDS-PAGE was used for the protein separation^58^, and cathode buffer (1 M Tris-Tricine pH 8.25, 1% SDS) and anode buffer (2 M Tris HCl, pH 8.9) were used for electrophoresis. Rat anti-hSeP mAbs (Clone AB1, AE2, AH5, BD1, BD3, BF2, DH9, AA3 and DC12)^59^, mouse anti-hSeP mAbs (Clone C18, C21 and C23) and rat anti-TrxR1 mAb KB12^60^ were used at 1 µg/mL for western blotting. Rabbit anti-hSeP pAb was prepared using purified hSeP as immunogen. We determined the cross-reactivity of AE2 and BD1 with mSeP; however, obvious immunoreactivity of these mAbs against mSeP was not observed. Reactivity of other mAbs against other species SeP was not evaluated. The following antibodies were used for western blotting: rabbit anti-GPx1 pAb (for mouse samples, ab22604; Abcam, Cambridge, MA, USA), mouse anti-GPx1 mAb (for human samples, clone GPX-347, M015-3; Medical Biological Laboratories, Nagoya, Japan), mouse anti-β-actin mAb (clone AC-15; Merck), mouse anti-GAPDH mAb (ab9484; Abcam), rabbit anti-phospho IR β (Tyr1146) pAb (#3021; Cell Signaling Technology, Beverly, MA, USA), rabbit anti-IR β pAb (#3027; CST), rabbit anti-phospho Akt (Ser473) pAb (#9271; CST), rabbit anti-Akt pAb (#9272; CST), chicken anti-insulin pAb (ab14042; Abcam), mouse anti-insulin mAb (clone L6B10, #8138; CST), and mouse anti-GFP mAb (Clone B-2, Sc-9996; Santa Cruz Biotechnology, Santa Cruz, CA, USA). The dilution of each Ab was determined according to the instruction. The full blot or gel corresponding to the main figures are shown in the Supplementary Figure as follows: Supplementary Fig. 11, Figs. 1d, e and 2a, b; Supplementary Fig. 12, Figs. 3e, 4a and 5a; Supplementary Fig. 13, Figs. 5c and 6b; Supplementary Fig. 14, Fig. 6d–f; Supplementary Fig. 15, Fig. 7b, g; Supplementary Fig. 16, Fig. 8a.

### Selenoprotein P uptake and Se-supply assay

SeP uptake and Se-supply activities were examined by the western blot analysis of hSeP and GPx1 levels in the whole lysate of treated cells, respectively. In the case of C2C12 cells, differentiation to myocytes were induced by DMEM containing 0.5% HS for 72 h, which resulted in the undetectable levels of SeP and GPx1. Differentiated C2C12 myocytes were treated with indicated concentration of hSeP or sodium selenite for 24 h, and then cells were washed and whole-cell lysates were subjected to western blotting. To assess the inhibitory activity of each mAb, hSeP (0.5 µg/mL) was incubated with each Ab (10 µg/mL) at room temperature for 2 h, and then this reaction mixture was added to differentiated C2C12 myocytes, and then cultured for 24 h. In the case of mSeP, mouse serum was incubated with mFHR pAb at room temperature for 2 h, and then this reaction mixture was added to differentiated C2C12 myocytes, and cultured for 24 h.

In the case of Jurkat cells, SeP uptake and Se-supply activities were examined by the western blot analysis of hSeP and GPx1 levels in the whole lysate of treated cells, respectively. Prior to this assay, Jurkat cells were cultured in the Se- and serum-free RPMI medium for 24 h, which resulted in the undetectable levels of SeP and GPx1. Serum- and Se-free Jurkat cells were treated with indicated concentration of hSeP or sodium selenite for 24 h, and then cells were washed and whole-cell lysates were subjected to western blotting. To assess the inhibitory activity of each mAb, hSeP (0.5 µg/mL) was incubated with each Ab (10 µg/mL) at room temperature for 2 h, and then this reaction mixture was added to serum- and Se-free Jurkat cells, and then cultured for 24 h.

### Transfection of small interfering RNA

The mouse LRP1-small interfering RNAs (siRNA) and the mouse ApoER2-siRNA were designed and manufactured by Dharmacon and Thermo Fisher Scientific, according to the current guidelines for effective knockdown by this method, respectively. The target sequences for mLRP1-siRNA (catalogue number D-040764-04-0050) and mApoER2-siRNA (catalogue number 5690369) were used. The siRNA were transfected into C2C12 cells by Lipofectamine RNAi MAX (Thermo Fisher Scientific). After transfection, myogenic differentiation of C2C12 cells was induced and used for further experiments. In the case of MIN6 cell, the siRNA were transfected into cells twice every second day by Lipofectamine RNAi MAX. MIN6 cells were used for further experiments 3 days after final transfection.

### Glutathione assay

Intracellular reduced glutathione (GSH) content was determined by using high-performance liquid chromatography conjugated with an electrochemical detector (ECD-100; Eicom). GSH content was calculated using reduced GSH as the standard. The results were shown as nmol GSH per mg of total protein.

### Animals

All animal experiments described in this study fully confirmed to the guidelines outlined in the Guide for the Care and Use of Laboratory Animals of Japan and were approved by the Animal Care Committee of the Doshisha University (approval no. A14032). Eight-week-old female C57BL/6J mice were obtained from Shimizu Laboratory Supplies (Kyoto, Japan). Five-week-old female KKAy mice were obtained from CLEA Japan (Tokyo, Japan). HFHSD (F2HFHSD) was purchased from Oriental Yeast (Tokyo, Japan). All animals were housed under a 12 h light/dark cycle and allowed free access to food and water.

### Injection of purified SeP and neutralizing Ab into mice

As described in Supplementary Fig. 4a, 9-week-old female C57BL/6J mice were injected twice with purified hSeP protein (1 mg/kg intraperitoneally ip)^16^. Control mice were injected ip with an identical volume of PBS (vehicle control). Treatment hSeP was made 12 and 2 h before tolerance tests or tissue sampling. Tissue samples were taken for western blotting after perfusion with saline. AE2 mAb (rat IgG1) and control IgG (20 mg/kg body weight) were administered 2 h before the first injection of SeP. Control rat IgG1 mAb (R&D systems) was used as a control.

Mouse models of diabetes were treated as described in Supplementary Fig. 8a. Nine-week-old male KKAy mice or 9-week-old male C57BL/6J mice fed HFHSD for 11 weeks were injected ip with mFHR pAb and control rabbit IgG (25 mg/kg body weight), and then underwent a glucose tolerance test (24 h after Ab injection), insulin tolerance test (48 h after Ab injection) and had tissue sampling (72 h after Ab injection). Control rabbit IgG (Thermo Fisher Scientific) was used as a control.

### Se assay

Levels of Se in serum and in tissues were determined according to the fluorometric method of Bayfield and Romalis^61^.

### Lipid analysis

Total cholesterol and triglyceride contents were determined by using cholesterol E-test wako and triglyceride E-test wako Kit (439–17501 and 432-40201; WAKO), respectively. The results were shown as mg of each lipid per g of total protein. The protein concentration was determined by using a bicinchoninic acid protein assay kit (Pierce Biotechnology) with BSA as the standard.

### Glucose and insulin tolerance test in mice

The study design is described in the scheme presented in Supplementary Figs. 4a and 8a. For glucose tolerance tests, mice were fasted for 12 h. After fasting, glucose was administered intraperitoneally, and blood glucose levels were measured at indicated times. The amount of glucose injected into C57BL/6J mice was 1.5 g/kg, while 0.3 g/kg glucose was injected into KKAy mice and mice fed an HFHSD. Blood glucose and insulin levels were determined using a glucose oxidase method (Glutest Sensor; Sanwa Kagaku, Kyoto, Japan) and Mouse Insulin ELISA Kit [T-type] (Shibayagi, Shibukawa, Japan), respectively. For insulin tolerance tests, mice were fasted for 4 h. Insulin (0.5 U/kg ip) was administered to C57BL/6J mice, while 10 U/kg insulin was administered ip to KKAy mice and mice fed an HFHSD. Blood glucose levels were measured at indicated times.

### Immunohistochemistry

The pancreas of treated mice were fixed in PBS containing 4% paraformaldehyde for 1 h, and then embedded in OCT compound (Sakura Finetek, Tokyo, Japan) and stored at −80 °C. The specimens were sectioned at 7 μm using a cryostat microtome. For morphometric analysis, three to four non-overlapping sections from each pancreas were used for each analysis. All analyses were conducted on at least three animals per treatment condition. To determine β-cell mass, sections were incubated with anti-insulin mAb (clone L6B10, #8138; CST), and then bound Abs were visualized with Alexa 568-conjugated anti-mouse IgG (Molecular Probes, Eugene, OR, USA). Hoechst 33342 dye (Dojindo, Kumamoto, Japan) was used to stain cell nuclei. Injected hSeP in the pancreas was visualized by using rat anti-hSeP mAb BD1 (5 µg/mL) and Alexa 488-conjugated anti-rat IgG (Molecular Probes). Anti-glucagon mAb (clone K79bB10, G2654; Sigma) and pAb (SAB4501137; Sigma) were used. The dilution of each Ab was determined according to the instruction. Cultured cells were fixed in PBS containing 4% paraformaldehyde for 1 h, and then immunostained. Specimens were observed using a laser-scanning confocal fluorescence microscope (LSM 710 ConfoCor 3; Carl Zeiss, Thornwood, NY, USA) equipped with Zeiss Efficient Navigation 2009 software. The total pancreatic area and insulin positive area of each section was measured using ImageJ software.

### Isolation of islets

Isolation of rat pancreatic islets was performed according to de Groot et al.^62^. Briefly, rat pancreas (Wister rat, male, 10 weeks old) were perfused and digested by collagenase, and then islets were handpicked and cultured in 24-well plates (10 islets/well) in RPMI-1640 culture medium supplemented with 10% FBS and antibiotics. After 2 h incubation, hSeP and selenocystine were added to isolated islets and incubated for 24 h. Treated cells were assayed for insulin release in vitro.

### In vitro insulin release assay

Insulin secretion analyses were performed according to Spegel et al^63^. Briefly, islets and MIN6 cells were preincubated for 30 min at 37 °C in a Krebs-Ringer bicarbonate buffer (KRB) composed of (in mM) 11.5 NaCl, 0.47 KCl, 0.12 KH_2_PO_4_, 0.12 MgSO_4_, 0.256 CaCl_2_, 20 NaHCO_3_, 10 HEPES, and supplemented with 5.6 mM glucose and 0.1% BSA (fatty acid free). Subsequently, islets and MIN6 cells were incubated at 37 °C for 1 h in KRB supplemented with 2.8 and 16.7 mM glucose, and then the supernatant was collected to determine insulin concentration using an ELISA.

### Epitope mapping

To map the epitope of each mAb, a series of expression constructs were generated. Fragments of human *SEPP1* were amplified by PCR and reinserted into the *Hin*dIII site of pEGFP-N1 (Invitrogen) using an In-Fusion HD Cloning Kit (Clontech TaKaRa Bio). Fragment number and amino acids are as follows: No. 1, Y60-S299; No. 2, Y60-V107; No. 3, S108-T155; No. 4, F156-P203; No. 5, F156-H217; No. 6, H204-R254; No. 7, H204-R261; and No. 8, D262-S299. Constructs were transfected into CHO cells using Lipofectamine 2000 (Invitrogen) transfection reagent. Cells were collected 48 h after initial transfection and lysed as described above. Total proteins (5–15 µg) were subjected to western blotting.

### Preparation of mFHR polyclonal antibody

Rabbit pAb against mFHR was prepared using a chemically synthesized peptide corresponding to the amino acid sequence around the FHR of mSeP (N-terminal 196-KTAEPSEVHSHHKHHNKHGC-C-terminal 215) as an immunogen. Keyhole limpet haemocyanin (KLH)-conjugated mFHR peptide was injected to rabbits, and then blood was collected. Obtained serum was fractionated by ammonium sulfate precipitation and then separated by affinity chromatography to mFHR peptide. Purified mFHR pAb was dialysed against PBS and used for further experiments after filter sterilization.

### Statistical analysis

Data are shown as the mean ± s.d. (in vitro) or s.e.m. (in vivo). Statistical analyses were performed using Excel software and IBM SPSS. The sample size was chosen based on trial experiments or experiments performed previously. In animal experiments, statistical methods were not used to determine sample size. We did not randomize animals, and we performed all experiments without blinding to the investigator, with the exception that the mice in the experiments with time-dependent change of blood mSeP levels in KKAy mice and HFHSD-fed mice were randomly assigned. All groups in the current experiments showed normal variance. Differences between the two groups were assessed using a two-tailed unpaired Student's *t-*test. Data involving more than two groups were assessed by analysis of variance (ANOVA) as described in the figure legends. *P* < 0.05 was considered to indicate significant differences.

### Data availability

All relevant data are available from the authors upon reasonable request.

## Electronic supplementary material

Supplementary Information
