# Supplementary material for: Selenoprotein P-neutralizing antibodies improve insulin secretion and glucose sensitivity in type 2 diabetes mouse models
Source: Nat Commun. 2017 Nov 21;8:1658. doi: 10.1038/s41467-017-01863-z (PMC5698464; doi:10.1038/s41467-017-01863-z)
Supplement: Supplementary file 1 — Supplementary Information [file 41467_2017_1863_MOESM1_ESM.pdf]

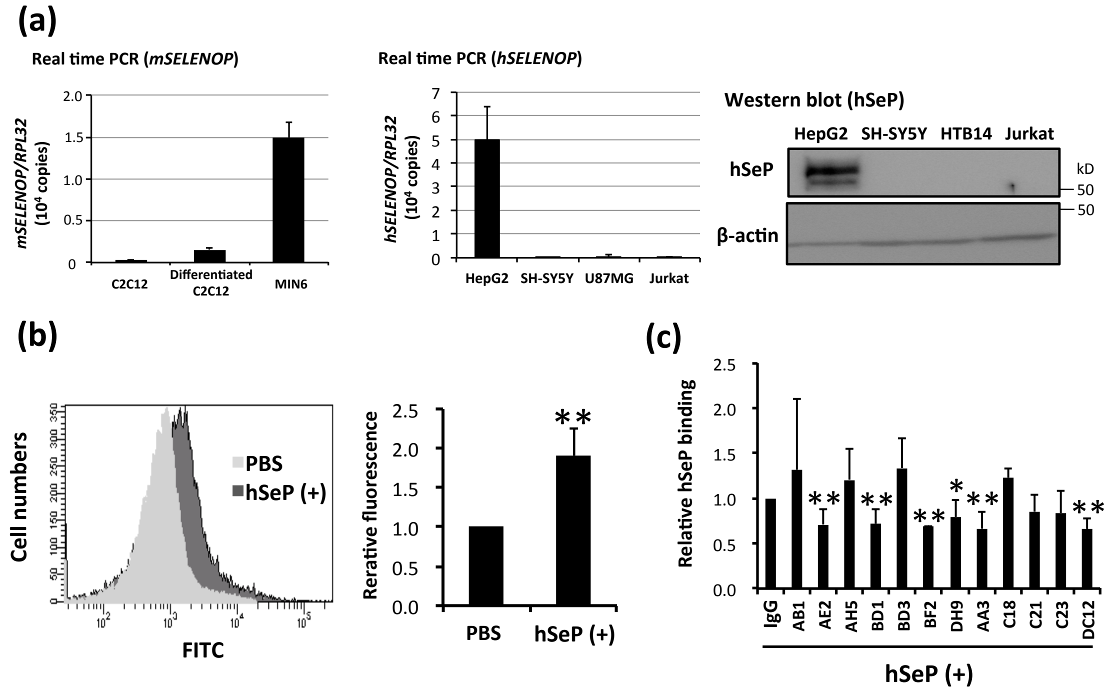

Supplementary Figure 1 | Relative expression level of SeP in cells and binding assay using Jurkat cells. (**a**) Relative mRNA levels of mouse and human selenoprotein P in cultured cells. Each cell was harvested for RNA isolation and protein extraction, and then analysed by real-time PCR and western blotting, respectively. The expression levels of *mouse selenoprotein P* (*mSELENOP,* left panel) and *human selenoprotein P* (*hSELENOP*, right panel) were normalized to that of *RPL32* mRNA (n = 4, means ± s.d.). (**b**) Human SeP interacting with the surface of Jurkat cells. Jurkat cells were incubated with human SeP (hSeP) at 4°C, and then hSeP on cell surface was determined by flow cytometry. Representative flow cytometry data and mean fluorescence intensity are shown (n = 6, means ± s.d.). ***P* < 0.01, Student *t* test. (**c**) Inhibitory effects of mAbs on the interaction between Jurkat cells and human SeP. Jurkat cells were incubated with hSeP in the presence of each mAb at 4°C, and then cells were analysed by flow cytometer analysis. Mean fluorescence intensity is shown (n = 3-5, mean ± s.d.). **P* < 0.05, ***P* < 0.01, vs control IgG, Tukey-ANOVA.

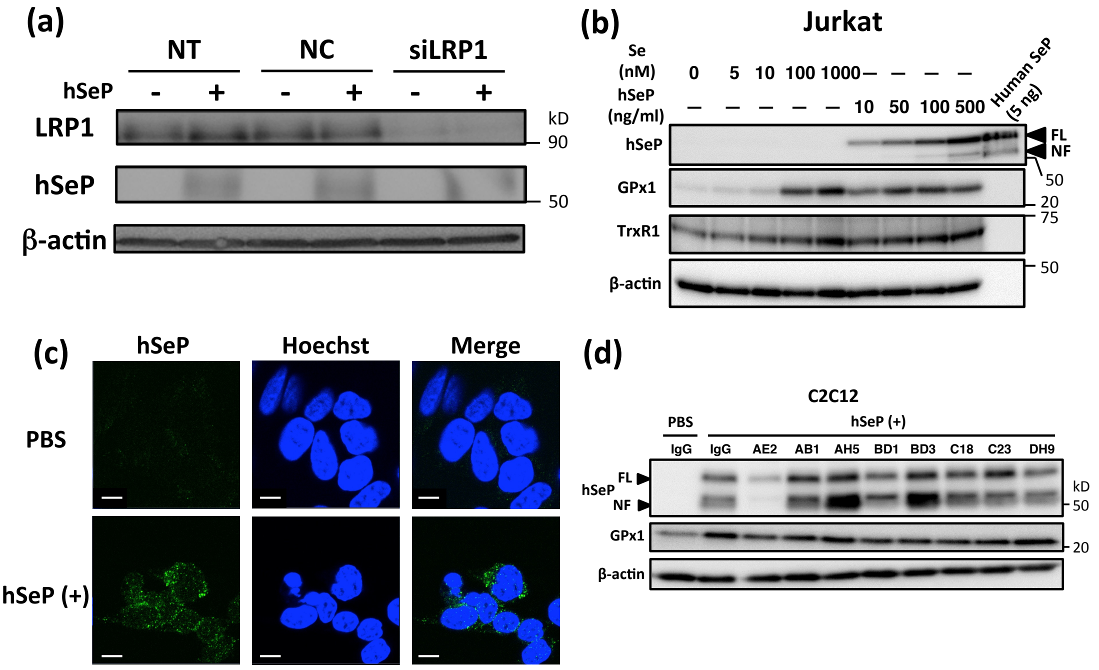

Supplementary Figure 2 | Analysis of cellular uptake of human SeP. (a) Effect of *LRP1*-siRNA on cellular uptake of human SeP in C2C12 myocytes. C2C12 cells were treated with *low-density lipoprotein receptor-related protein 1* (*LRP1*)-siRNA (*siLRP1*) or non-specific RNA (negative control, NC), myogenic differentiation was induced, and then the cells were treated with human SeP (hSeP, 10 μg/ml) for 48 h. Whole cell lysate was analysed by western blotting. NT: non transfection. **(b)** Cellular uptake and Se-supply activity of human selenoprotein P in Jurkat cells. Cells were treated with each concentration of sodium selenite (Se) and hSeP in Se- and serum-deficient medium for 24 h, as described in the Methods, and then whole-cell lysates were analysed by western blotting. GPx1, glutathione peroxidase 1; TrxR1, thioredoxin reductase 1. **(c)** Immunohistochemical analysis of human selenoprotein P-treated Jurkat cells. Cells were treated with hSeP (0.5 μg/mL) in Se- and serum-deficient medium for 24 h. Treated cells were immunostained with a mAb against hSeP and then visualized using fluorescence confocal microscopy. Scale bars = 5 μm. **(d)** Screening of neutralizing antibody for Se-supply activity of SeP in C2C12 myocytes. Cells were treated with hSeP (0.5 μg/mL) in the presence of each mAb (10 μg/mL) in culture medium containing 0.5% horse serum for 24 h. Cellular hSeP and GPx1 levels in whole-cell lysates were determined by western blotting.

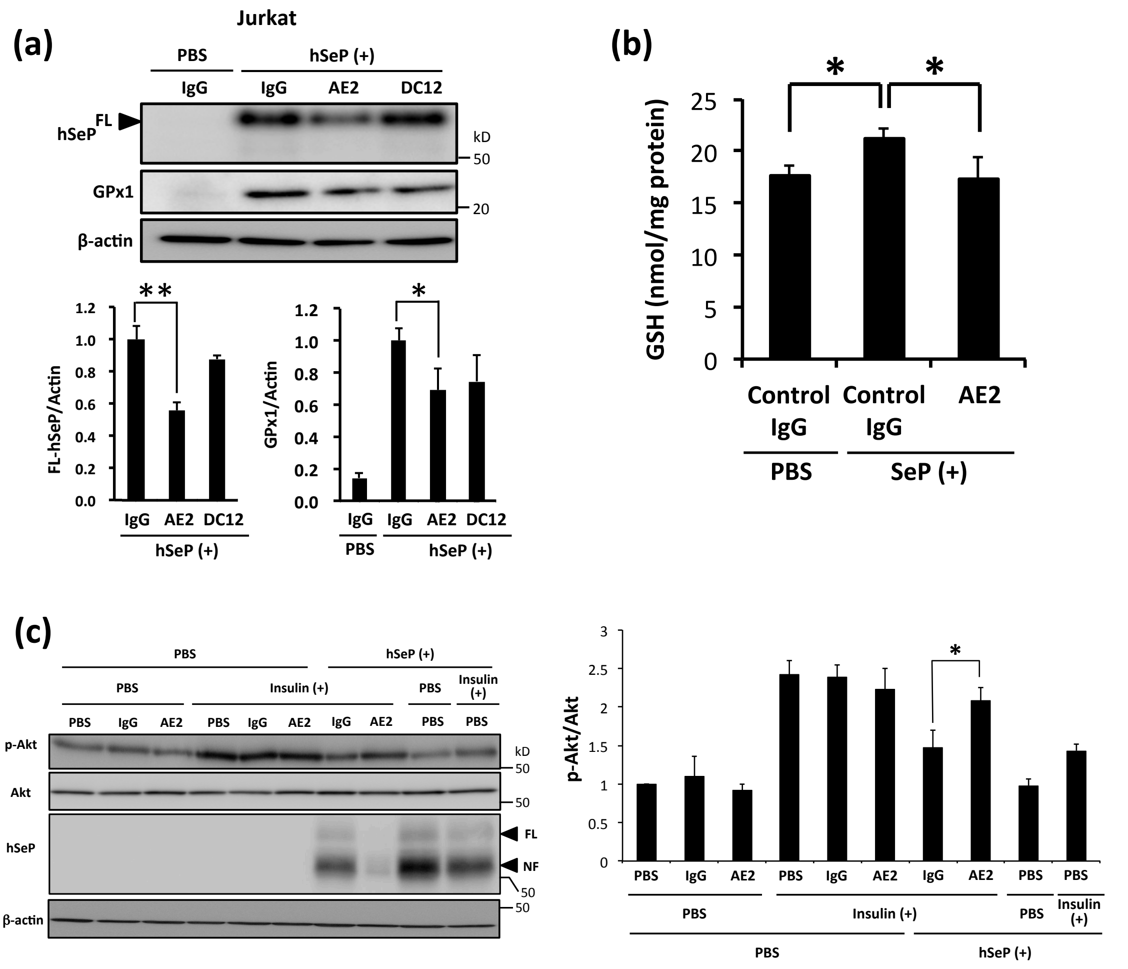

Supplementary Figure 3 | Effects of binding-inhibitory Abs for Se-supply activity of SeP. (**a**) Effects of binding-inhibitory Abs for Se-supply activity of SeP in Jurkat cells. Cells were treated with human SeP (hSeP, 0.5 μg/mL) in the presence or absence of each mAb (10 μg/mL) in Se- and serum-deficient medium for 24 h. Human SeP and GPx1 levels in whole-cell lysates were determined by western blotting (n = 3, means ± s.d.). The intensity of the hSeP band was only determined in the hSeP-treated group. In the case of GPx1, the same area of each lane was determined. $^{**}P < 0.01$, $^{*}P < 0.05$, Tukey-ANOVA. (**b**) Effects of binding-inhibitory Ab on cellular redox status in C2C12 myocytes. C2C12 myocytes were treated with 10 μg/mL hSeP in the presence of AE2 mAb or control IgG (500 μg/mL) for 72 h. Reduced glutathione (GSH) in the treated cells was assayed (n = 3, means ± s.d.). $^{*}P < 0.05$, Tukey-ANOVA. (**c**) Effects of binding-inhibitory Ab on insulin-induced Akt phosphorylation. C2C12 myocytes were treated with 10 μg/mL hSeP in the presence of AE2 mAb or control IgG (500 μg/mL)

for 72 h. Treated cells were stimulated by insulin (100 ng/mL), and then analysed by western blotting (n = 3, means ± s.d.). $^{*}P < 0.05$, Tukey-ANOVA.

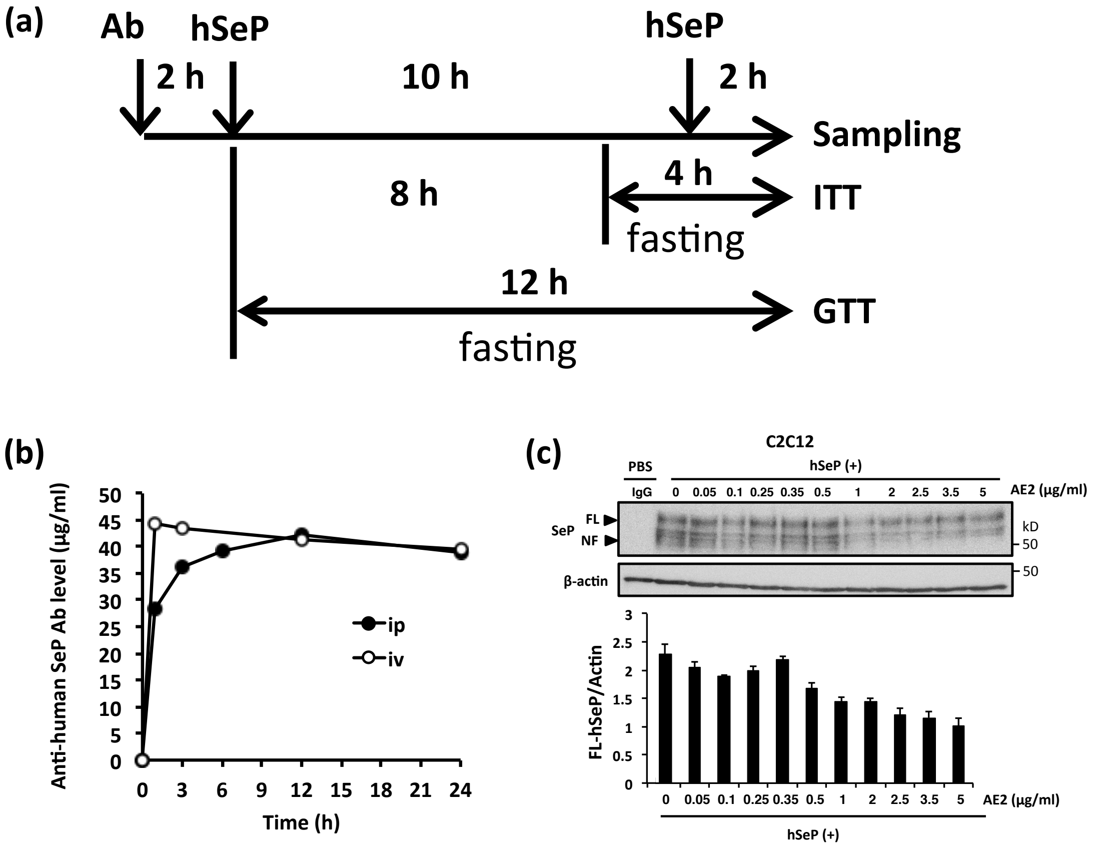

Supplementary Figure 4 | Scheme for SeP administration in mice and concentration-dependent effects of AE2. (a) Experimental scheme for administration of selenoprotein P and antibodies. C57BL/6J mice (female, 9 weeks old) were injected with antibody (Ab, 20 mg/kg intraperitoneally (ip)) and human SeP (hSeP, 1 mg/kg ip), and subjected to western blot analysis, an insulin tolerance test (ITT), and glucose tolerance test (GTT), as described in the Methods. Duration of Fasting for each test is shown. **(b)** Evaluation of blood levels of anti-human selenoprotein P antibody. C57BL/6J mice (female, 9 weeks old) were injected intraperitoneally (ip) or intravenously (iv) with anti-human SeP antibody (1 mg/kg), and blood levels of injected antibody were determined by direct ELISA, as described in the Methods. Closed circle, ip; open circle, iv. **(c)** Concentration-dependent effects of AE2 on hSeP incorporation. C2C12 myocytes were incubated with 0.5 μg/mL hSeP in the presence of indicated amounts of AE2 for 24 h, and then whole-cell lysates were analysed by western blotting (n = 3, means ± s.d.).

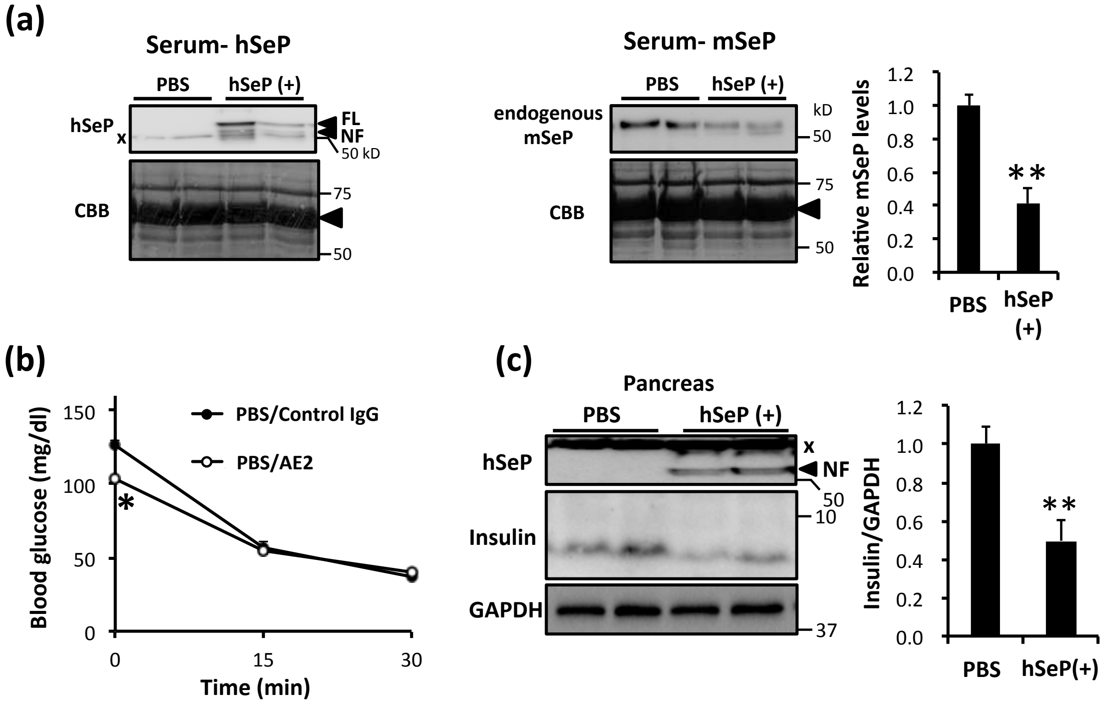

Supplementary Figure 5 | Effects of human SeP and AE2 administration on C57BL/6J mice. **(a)** Effects of human SeP administration on endogenous mouse SeP. Mouse serum was collected 2 h after the second human SeP (hSeP) injection following the scheme of sampling shown in Supplementary Fig. 4a under fed conditions, and then serum hSeP and endogenous mouse SeP (mSeP) were determined by western blotting. Graphs display the results of densitometric quantification, normalized to major protein (albumin, indicated by black arrowhead) stained with CBB (n = 5, means ± s.e.m.). $^{**}P < 0.01$, Student *t* test. **(b)** Evaluation of blood glucose levels in ITT in a group administered with Ab and PBS. C57BL/6J mice (female, 9 weeks old) were injected ip with AE2 mAb or control IgG following the scheme of insulin tolerance test in Supplementary Fig. 4a, and then insulin (0.5 U/kg) was administered and blood glucose determined (n = 5-8, means ± s.e.m.). Closed circle, PBS/control IgG; open circle, PBS/AE2. $^{*}P < 0.05$, Tukey-ANOVA. **(c)** Effects of human selenoprotein P treatment on insulin levels in the pancreas. C57BL/6J mice (female, 9 weeks old) were injected twice with human SeP (1 mg/kg ip) or PBS control 12 and 2 h before sampling as described in Supplementary Fig. 4a. After perfusion with saline, extracted pancreas tissues were homogenized and protein samples were analysed by western blotting (n = 4-5, means ± s.e.m.). $^{**}P < 0.01$, Student *t* test.

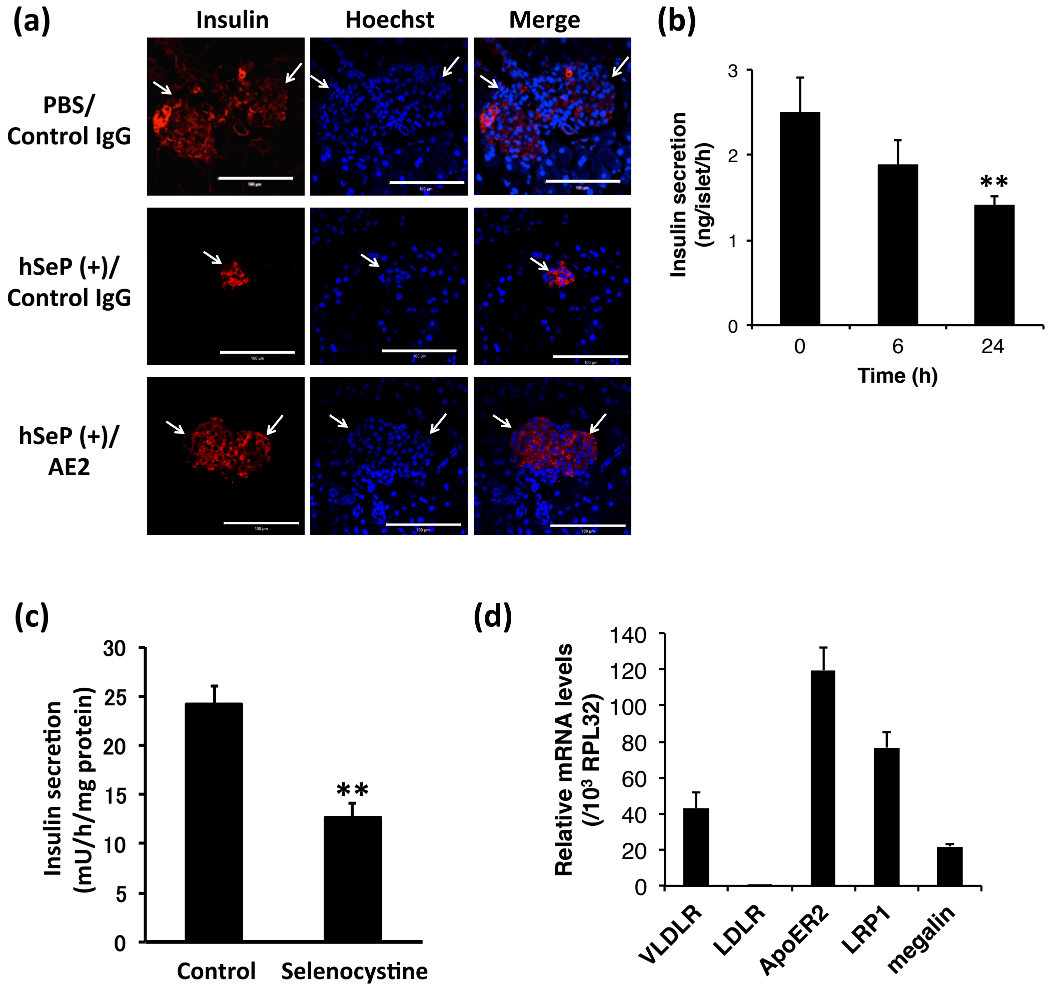

Supplementary Figure 6 | Effects of excess SeP and selenocystine on pancreatic β cells and its model. (a) AE2 rescued hSeP-mediated reduction in pancreatic β cell area. Pancreas tissues from AE2- and hSeP-treated mice were examined immunohistochemically using anti-insulin Ab. Scale bars = 100 μm. **(b)** Time-dependent effect of selenocystine on insulin secretion by isolated rat islet. Isolated rat islets were treated with 800 nM selenocystine for the indicated times, and then insulin secretion induced by high glucose was evaluated as described in the Methods (n = 3-4, means ± s.d.). Value of selenocystine was calculated by using the previous determinants of purified hSeP, namely 6.3 selenocysteines for a polypeptide mass of 41 kDa (as estimated from the sums of amino acid composition)[1]. $^{**}P < 0.01$, vs time 0, Tukey-ANOVA. **(c)** Effect of selenocystine on insulin secretion by MIN6 cells. MIN6 cells were treated with 800 nM selenocystine for 24 h, and then insulin secretion induced by high glucose was determined (n = 3, means ± s.d.). $^{**}P < 0.01$, Student *t* test. **(d)** Relative mRNA levels of lipoprotein receptors in MIN6 cells. MIN6 cells were

harvested for RNA isolation and real-time PCR analysis. The expression levels of each *lipoprotein receptor* were normalized to that of *RPL32* mRNA (n = 4, means ± s.d.).

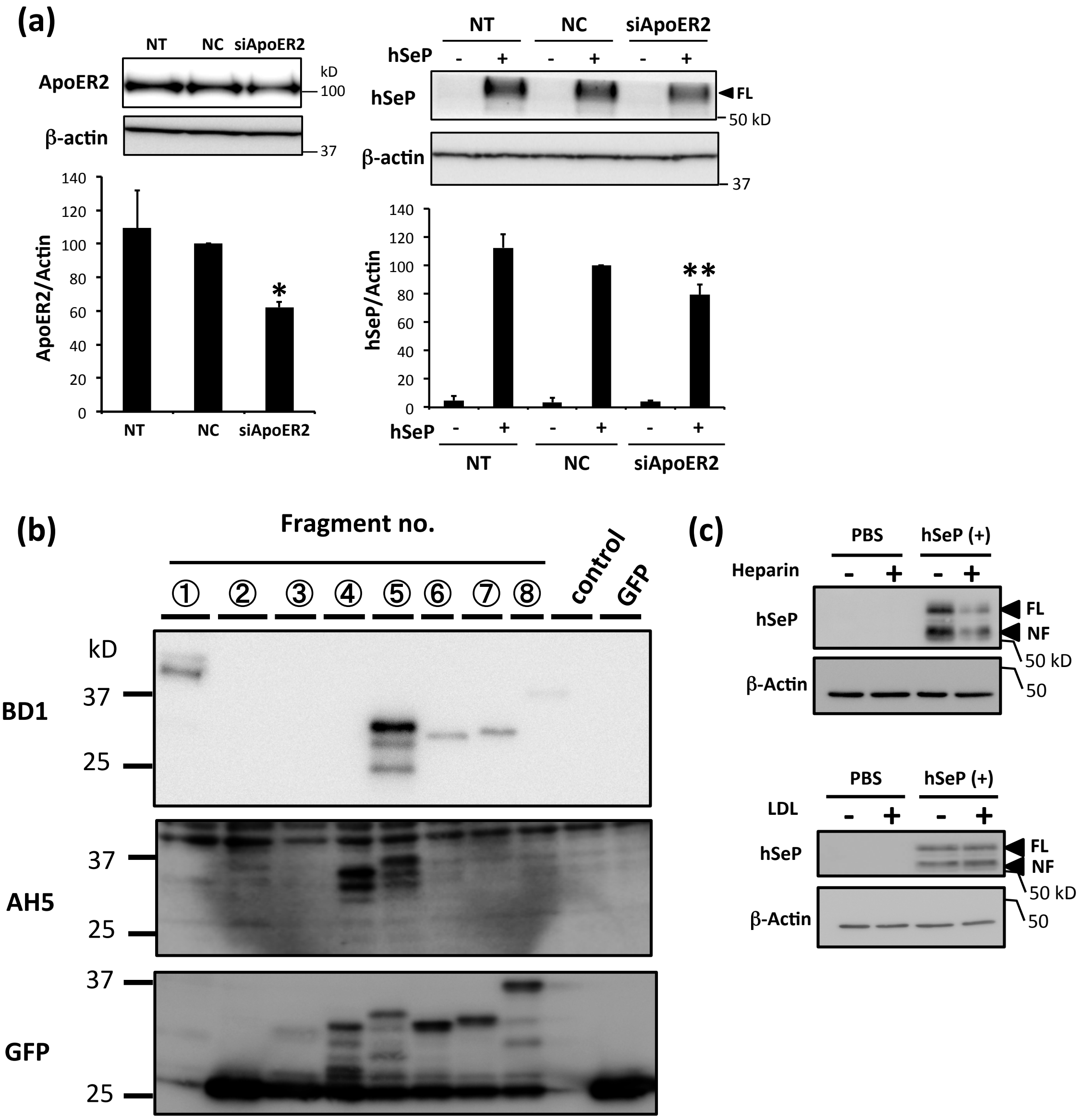

Supplementary Figure 7 | Analysis of SeP uptake in cultured cells and identification of epitopes. (a) Effect of ApoER2-siRNA on cellular uptake of human SeP in MIN6 cells. MIN6 cells were treated with ApoER2-siRNA (siApoER2) or non-specific RNA (negative control, NC) twice for 6 days, and whole cell lysate was subjected to western blotting using anti-ApoER2 Ab (n = 3, means ± s.d., left panel). MIN6 cells were pretreated with ApoER2-siRNA for 6 days and then treated with hSeP (0.1 μg/mL) for 24 h. Whole cell lysate was analysed by western blotting (n = 3, means ± s.d., right panel). *P < 0.05, **P < 0.01, Tukey-ANOVA, when compared with negative control. NT: non transfection. **(b)** Identification of epitopes recognized by antibodies to human selenoprotein P. Whole-cell lysates transfected with GFP-tagged

deletion construct of human SeP were analysed by western blotting using anti-human SeP mAb BD1 and AH5. **(c)** Effects of heparin and LDL on the cellular uptake of SeP by C2C12 myocytes. C2C12 myocytes were treated with hSeP (0.5 μg/mL) in the presence of heparin (1 mg/mL, left panel) or low-density lipoprotein (LDL, 100 μg/mL, right panel) for 24 h at 37°C. Whole-cell lysates were analysed by western blotting.

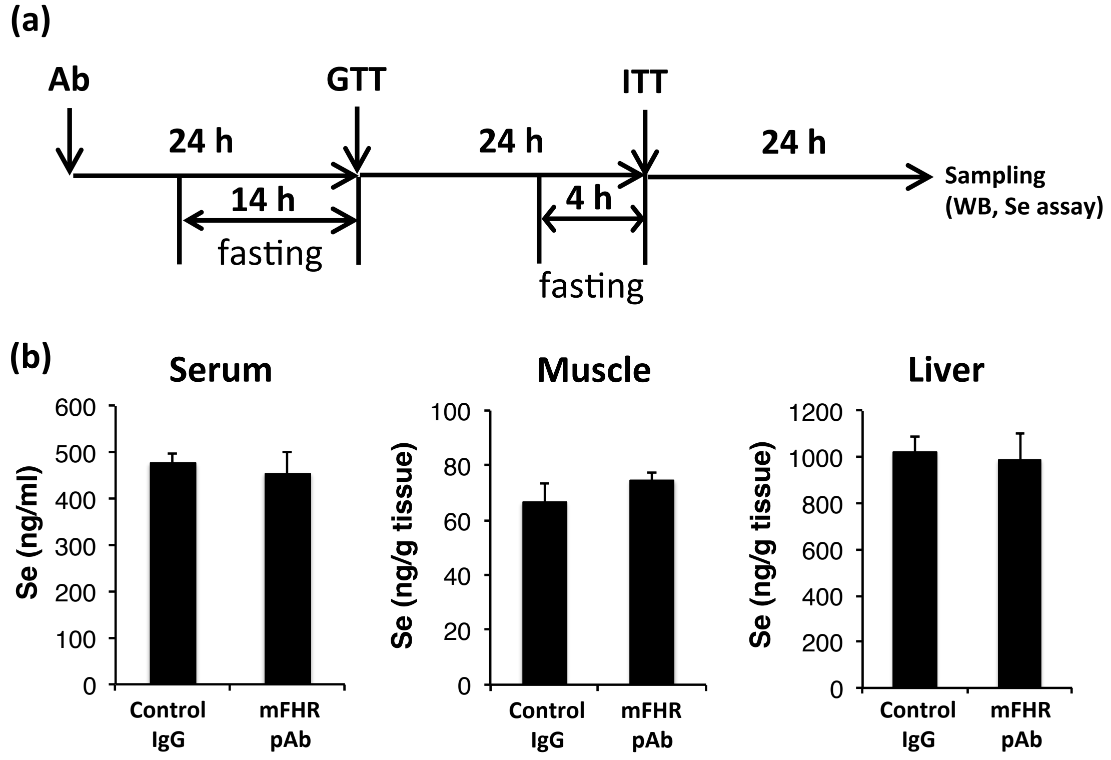

Supplementary Figure 8 | Scheme for Ab administration in diabetes model mice and Se contents in Ab-treated KKAy mice. (a) Scheme for administration of antibodies to mice. KKAy mice (male, 9 weeks old) or C57BL/6J mice (male, 9 weeks old) fed a high-fat, high-sucrose diet for 11 weeks were injected intraperitoneally with antibody (Ab, 25 mg/kg), and underwent a glucose tolerance test (GTT), insulin tolerance test (ITT), and had tissue sampling for western blotting (WB) or Se assay, as described in the Methods. Duration of Fasting for each test is shown. **(b)** Se contents in KKAy mice treated with mouse SeP-neutralizing Ab. Mouse SeP-neutralizing mFHR pAb or control IgG (25 mg/kg) was administered 72 h before sampling, as described in the scheme of Supplementary Fig. 8a (n = 5, means ± s.e.m.). Tissue samples were taken for selenium assay after perfusion of saline.

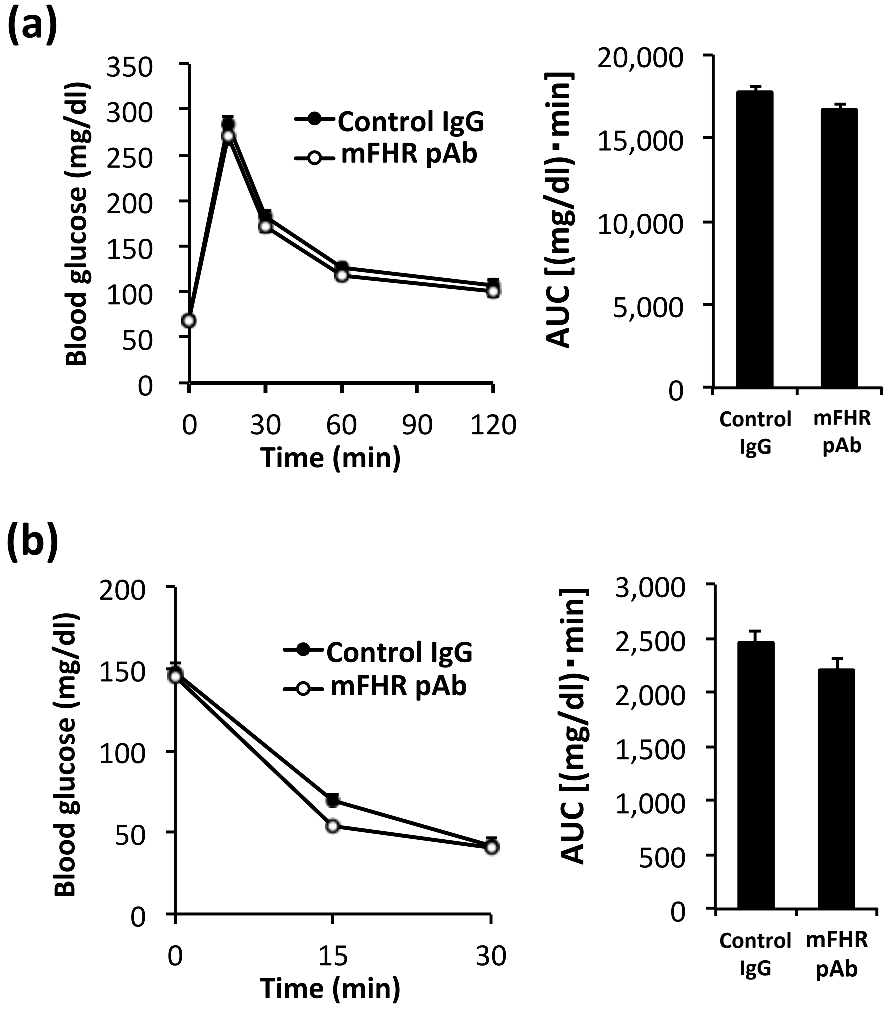

Supplementary Figure 9 | Effects of mSeP-neutralizing Ab on glucose and insulin tolerance in normal mice. (**a**) Effects of mSeP-neutralizing Ab on glucose tolerance in normal C57BL/6J mice. Mouse SeP-neutralizing mFHR pAb or control IgG (25 mg/kg) was administered following the scheme of the glucose tolerance test described in Supplementary Fig. 8a (n = 5, means ± s.e.m.). Closed circle, Control IgG; open circle, mFHR pAb. Glucose (1.5 g/kg) was administered intraperitoneally, and blood glucose was determined as indicated. The area under the curve for blood glucose levels is also shown (n = 5, means ± s.e.m.). No significant difference was observed between the groups. (**b**) Effects of mSeP-neutralizing Ab on insulin tolerance in normal C57BL/6J mice. Mouse SeP-neutralizing mFHR pAb or control IgG (25 mg/kg) was administered following an insulin tolerance test (n = 5, means ± s.e.m.). Insulin (0.5 U/kg) was administered, and blood glucose was determined. Closed circle, Control IgG; open

circle, mFHR pAb. The area under the curve for blood glucose levels is shown (n = 5, means ± s.e.m.). No significant difference was observed between the groups.

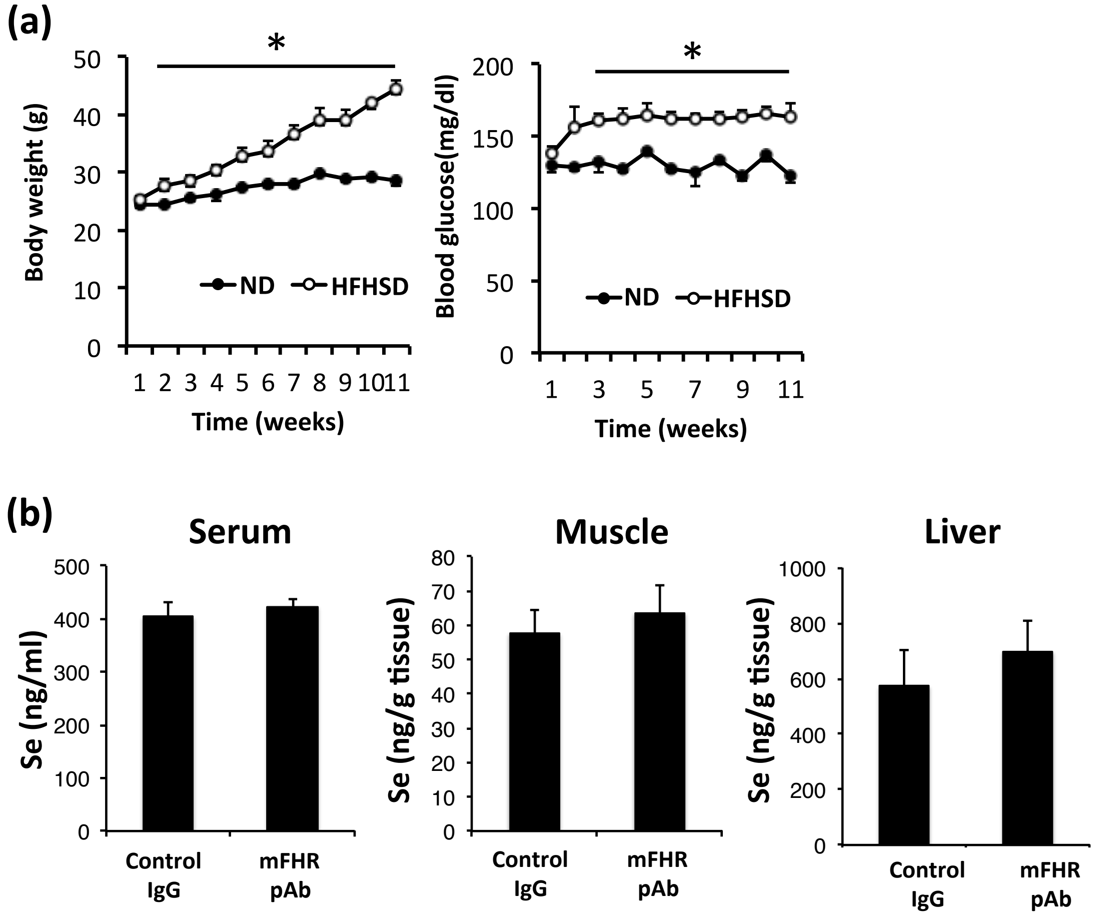

Supplementary Figure 10 | Body weight, blood glucose, and Se content in mice fed a HFHSD. **(a)** Time-dependent change of body weight and blood glucose in mice fed a HFHSD. Body weight and blood glucose levels were determined in mice fed a high-fat, high-sucrose diet (HFHSD) or normal diet (ND). Significant difference was observed from 2 weeks and 3 weeks, respectively (n = 3, means ± s.e.m.). Closed circle, ND; open circle, HFHSD. *$P < 0.05$, Tukey-ANOVA. **(b)** Se contents in mice fed a HFHSD treated with mouse SeP-neutralizing Ab. Mouse SeP-neutralizing mFHR pAb or control IgG (25 mg/kg) was administered following the sampling scheme in Supplementary Fig. 8a (n = 5, means ± s.e.m.). Tissue samples were taken for selenium assay after perfusion of saline.

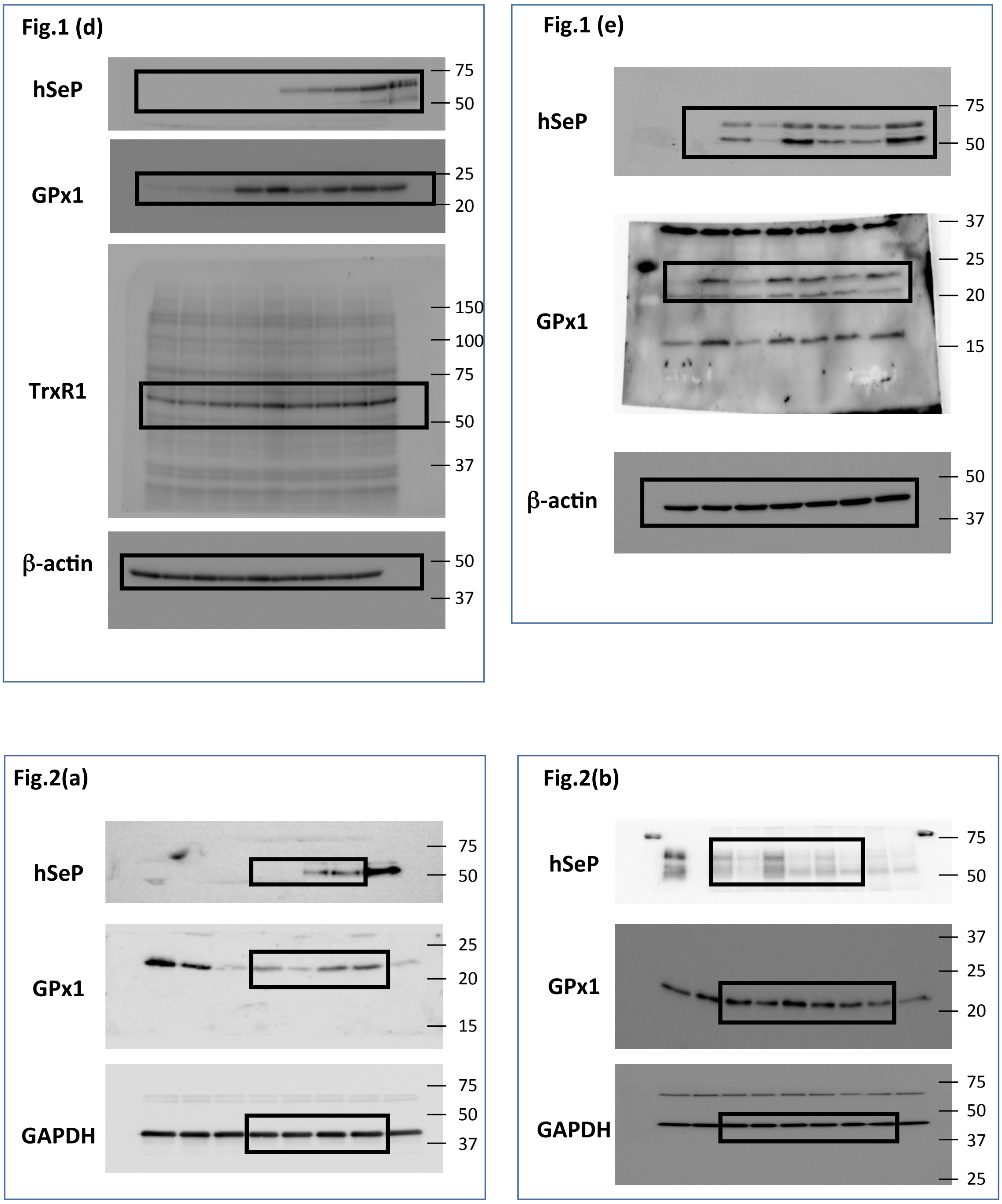

Supplementary Figure 11 | The full blot or gel corresponding to main figures as indicated. Used area is enclosed.

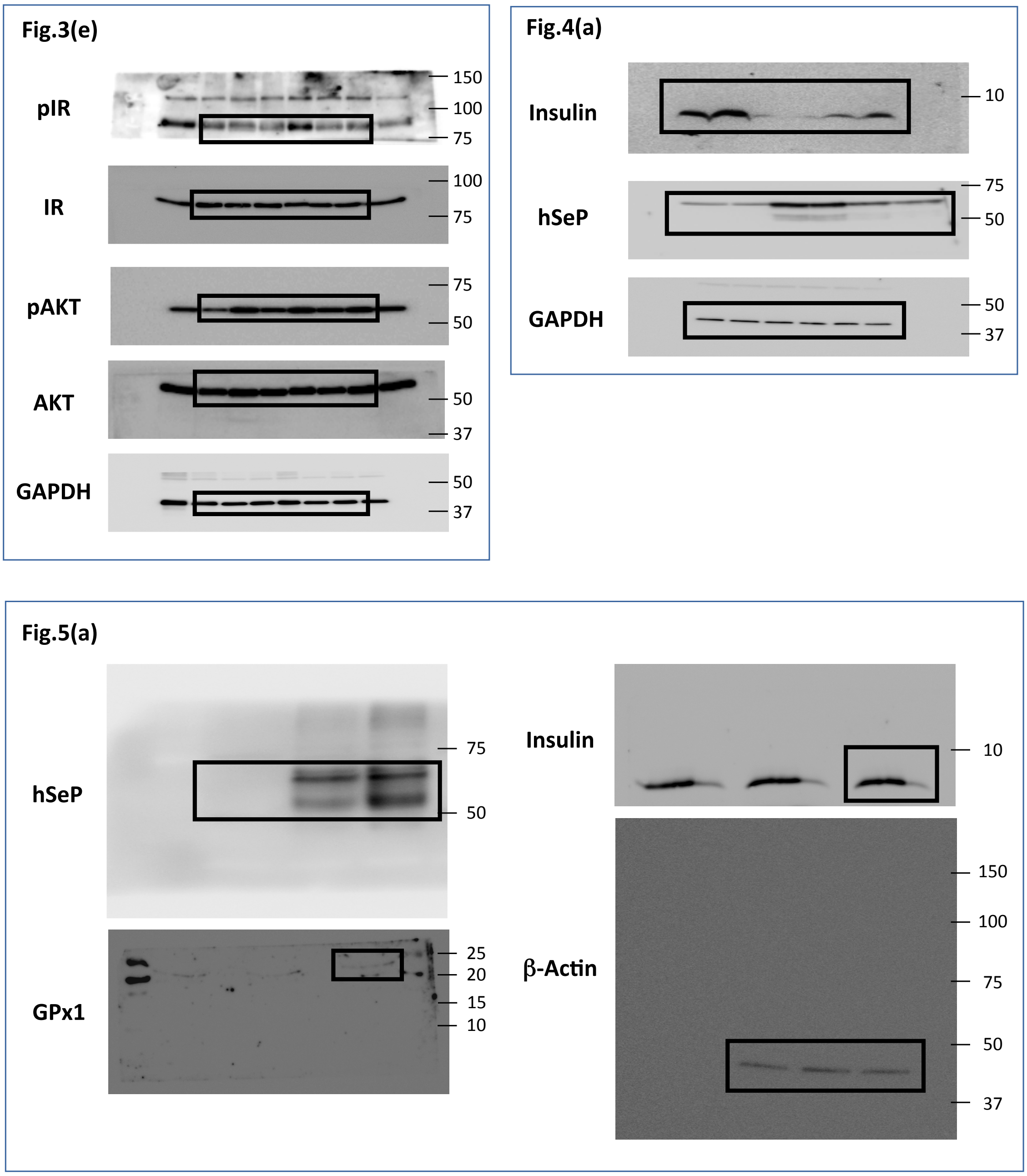

Supplementary Figure 12 | The full blot or gel corresponding to main figures as indicated. Used area is enclosed.

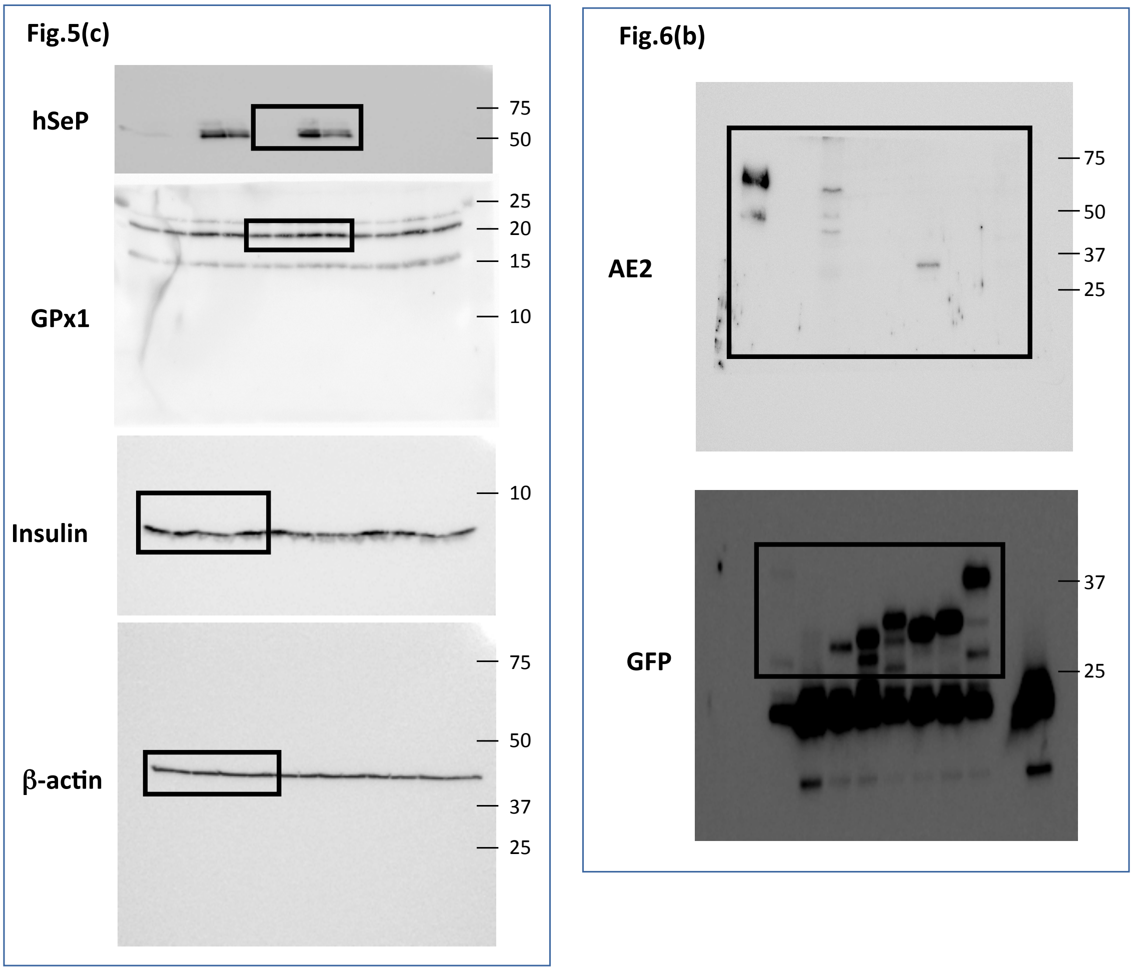

Supplementary Figure 13 | The full blot or gel corresponding to main figures as indicated. Used area is enclosed.

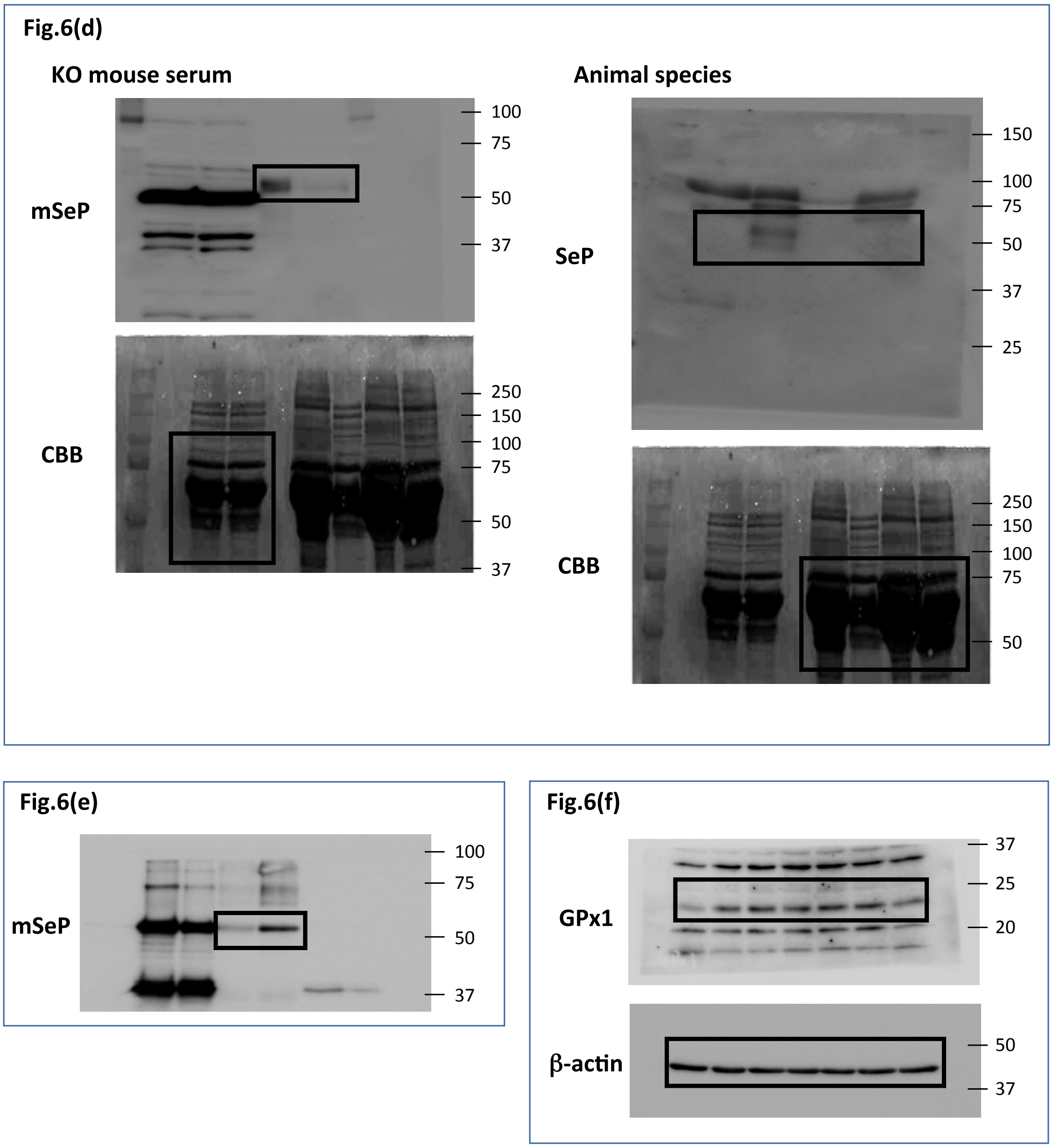

Supplementary Figure 14 | The full blot or gel corresponding to main figures as indicated. Used area is enclosed.

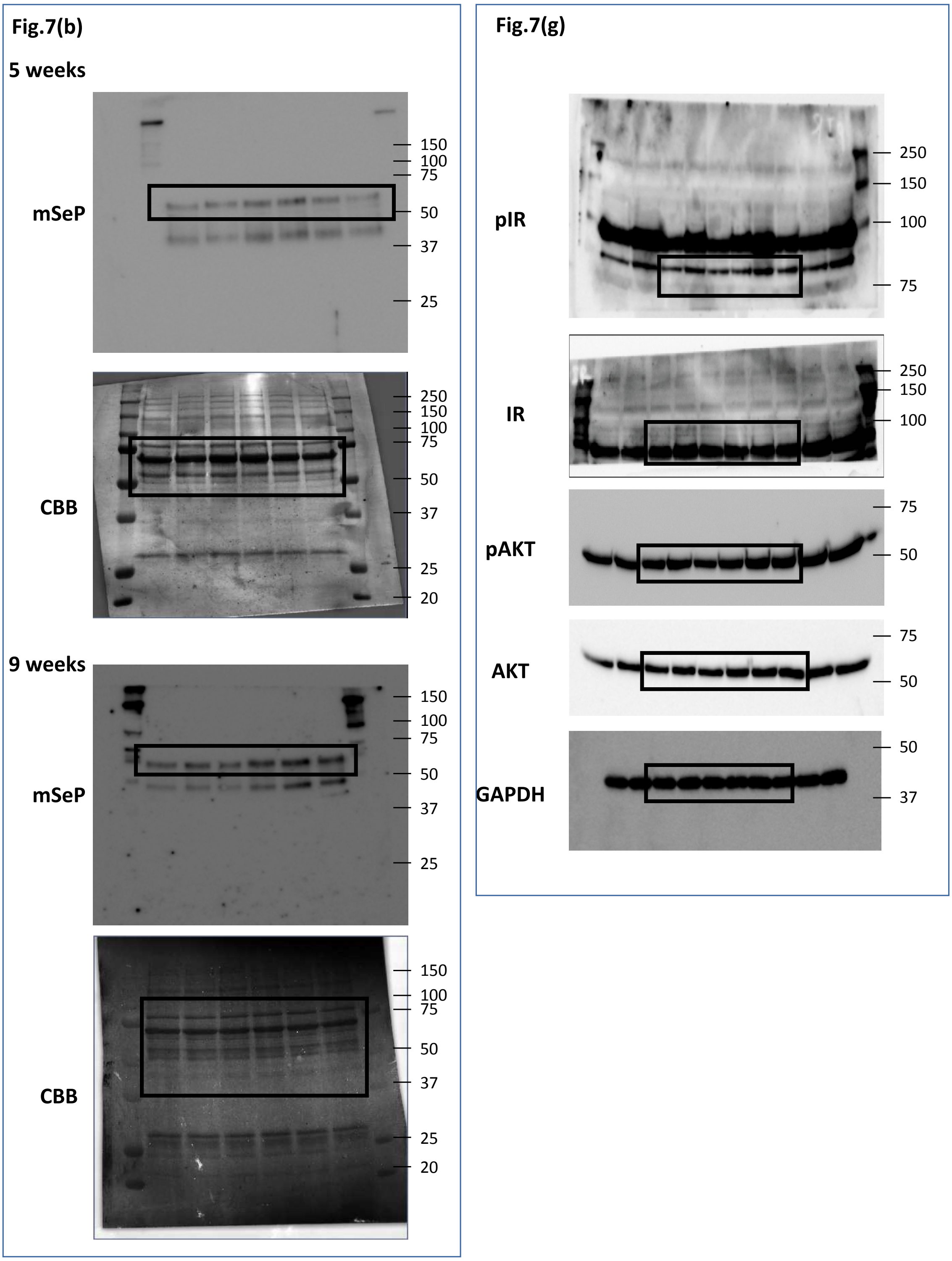

Supplementary Figure 15 | The full blot or gel corresponding to main figures as indicated. Used area is enclosed.

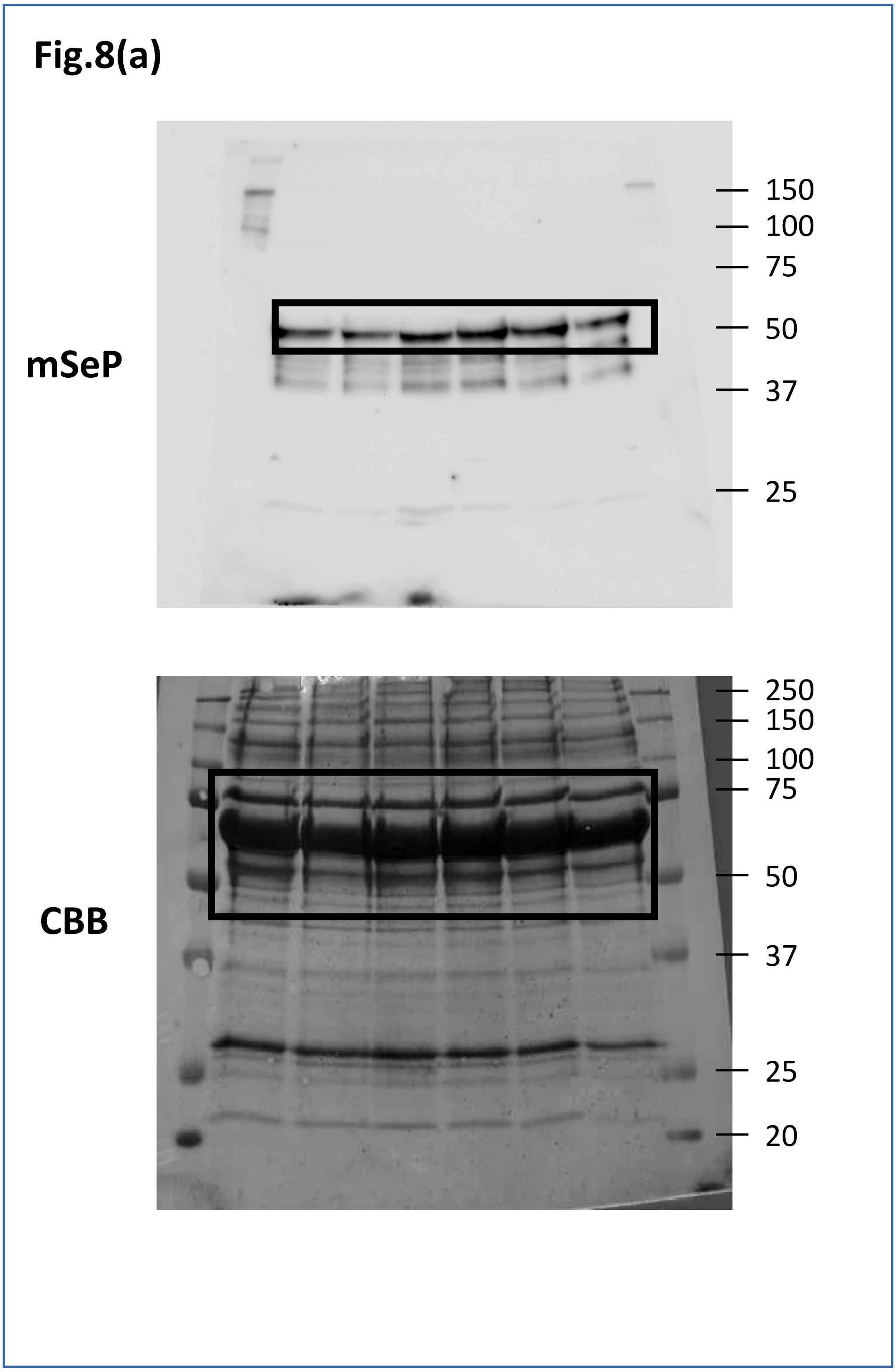

Supplementary Figure 16 | The full blot or gel corresponding to main figures as indicated. Used area is enclosed.

Supplementary Table 1 Comparison of lipid contents in liver of hSeP-injected mice[a]

	PBS		hSeP (+)	
	IgG	AE2	IgG	AE2
Total cholesterol (mg/g protein)	3.6 ± 0.3	3.6 ± 0.5	3.6 ± 0.4	3.8 ± 0.2
Triglyceride (mg/g protein)	19.5 ± 2.0	18.4 ± 0.9	19.0 ± 4.3	19.3 ± 3.4

[a] Following the scheme of Supplementary Fig. 4a, C57BL/6J mice (female, 9 weeks old) were injected with AE2 mAb or control IgG (20 mg/kg) and purified hSeP (1 mg/kg) or control PBS. After perfusion of saline, extracted liver tissues were homogenized and subjected to the lipid analysis, as described in the Methods (n = 4-5, means ± s.e.m.). No significant difference between these groups was observed.

Supplementary Table 2 Comparison of lipid contents in liver of KKAy mice treated with mSeP-neutralizing Ab[a]

	KKAy	
	IgG	mFHR pAb
Total cholesterol (mg/g protein)	32.2 ± 5.7	19.4 ± 1.6*
Triglyceride (mg/g protein)	55.0 ± 6.5	41.3 ± 4.0

[a] Mouse SeP neutralizing mFHR pAb or control IgG (25 mg/kg) was administrated 72 hr before sampling, as described in the scheme of Supplementary Fig. 8a (n = 4, means ± s.e.m.). Tissue samples were taken for lipid assay after perfusion of saline. *p<0.05, Student t test.

Supplementary Table 3 Comparison of lipid contents in liver of mice fed a high-fat, high-sucrose diet treated with mSeP-neutralizing Ab[a]

	HFHSD fed mice	
	IgG	mFHR pAb
Total cholesterol (mg/g protein)	16.7 ± 2.8	13.8 ± 2.7
Triglyceride (mg/g protein)	34.2 ± 6.2	25.2 ± 3.9

[a] Mouse SeP neutralizing mFHR pAb or control IgG (25 mg/kg) was administrated 72 hr before sampling, as described in the scheme of Supplementary Fig. 8a (n = 5, means ± s.e.m.). Tissue samples were taken for lipid assay after perfusion of saline. Significant difference between these groups was not observed.

SUPPLEMENTARY REFERENCES:

1. Saito, Y. *et al*. Selenoprotein P in human plasma as an extracellular phospholipid hydroperoxide glutathione peroxidase. Isolation and enzymatic characterization of human selenoprotein p. *J. Biol. Chem.* **274**, 2866-2871 (1999).
